# Multi-stage convo-enhanced retinex canny DeepLabv3+ FusionNet for enhanced detection and classification of bleeding regions in GI tract

**DOI:** 10.1038/s41598-025-24716-y

**Published:** 2025-11-20

**Authors:** V. Sharmila, S. Geetha

**Affiliations:** School of Computer Science and Engineering, Vellore Institute of Technology, Chennai Campus, Vandalur – Kelambakkam, Chennai, 600127 Tamilnadu India

**Keywords:** GI tract bleeding, Clip-BiRetinexnet, Color enhancement, Edge detection, Medical image processing, Computational biology and bioinformatics, Health care

## Abstract

Detection of gastrointestinal bleeding in Wireless Capsule Endoscopy (WCE) images and accurate bleeding region segmentation and classification is crucial for exact diagnosis and treatment, as early detection can prevent severe complications. However, it remains challenging due to the inability of current methods to effectively differentiate between types of bleeding and handle complex borders of lesions. In this paper, a new framework: Multi-Stage Convo-Enhanced Retinex Canny DeepLabV3+ FusionNet is proposed to better tackle these challenges. Existing feature extraction algorithms struggle with colour differentiation and texture recognition, often failing to miss fine-scale textures that distinguish active bleeding from coagulated blood effectively. Hence, this approach is initiated with Clip-BiRetinexNet for preprocessing, enhancing image contrast and color consistency using Clipped Histogram Equalization and Bilateral Filtered Retinex thereby capturing fine-scale textures that distinguish active bleeding from coagulated blood. Existing segmentation and classification methods struggle with irregular and complex borders of bleeding types like ulcers and vascular lesions due to ineffective border detection. Therefore, the segmentation in this proposed model is handled by Hough Canny-Frangi Enhanced DeepLabV3+, improving edge detection and vascular pattern enhancement to delineate accurately irregular lesions. Next, a ResNet-NaiveBayes Fusion was shown for classification, offering effective probabilistic classification. The implementation results show that the proposed approach outperforms the state-of-the-art methods with a high mean pixel accuracy of 97.6%, classification accuracy of 99.2% and Dice Similarity Coefficient of 99.6%.

## Introduction

Detecting gastrointestinal bleeding in WCE involves leveraging advanced image processing and machine learning techniques to analyze video frames captured by the capsule as it traverses the digestive tract^1^. WCE offers a non-invasive method to visualize the small intestine, where bleeding can be challenging to detect due to its intermittent nature and variable presentation. Current research focuses on developing algorithms that can segment bleeding regions from normal tissue, classify bleeding types (such as ulcers, angiodysplasia, or varices), and assess the severity and location of bleeding events^2^. Identifying gastrointestinal (GI) tract bleeding involves pre-processing WCE images to enhance quality, followed by extracting features like texture, colour, and geometric descriptors to differentiate bleeding from normal tissue. These features are then used in supervised machine learning algorithms such as SVMs or CNNs to classify bleeding types (e.g., ulcers, angiodysplasia)^3^. Post-classification, localization techniques pinpoint bleeding locations, and severity assessments gauge the extent of bleeding, aiding clinical decisions. Integrated into decision support systems, these classifications help clinicians interpret findings and plan interventions effectively, improving patient care in GI health management^4^.

In the feature extraction stage of gastrointestinal bleeding detection in Wireless Capsule Endoscopy, several key steps are employed to capture relevant information from the captured video frames^5^. Initially, pre-processing techniques such as colour normalization and noise reduction are applied to enhance image quality and mitigate artifacts. Subsequently, feature extraction focuses on extracting discriminative features that distinguish bleeding regions from normal tissue. This typically involves the computation of texture descriptors (e.g., Haralick features, Gabor filters) to capture spatial patterns and structural information within the images^6^. Additionally, colour-based features such as histogram-based colour moments or colour channel statistics are computed to characterize the chromatic properties of bleeding areas. Spatial features, including shape descriptors and geometric attributes, further contribute to differentiating between bleeding types (e.g., ulcers, angiodysplasia)^7^. These extracted features serve as input to machine learning algorithms, such as support vector machines (SVMs) or deep neural networks, for subsequent classification and localization of bleeding instances within the WCE imagery^8^.

Segmentation and classification in gastrointestinal (GI) tract bleeding disease involve intricate processes to effectively identify and categorize bleeding regions within WCE imagery. Segmentation techniques are crucial initial steps, employing image processing algorithms to delineate bleeding areas from surrounding normal tissue^9^. These methods typically utilize advanced approaches such as deep learning-based semantic segmentation or region-based methods to accurately outline bleeding regions based on color intensity, texture patterns, and spatial relationships. Once segmented, classification algorithms come into play, utilizing extracted features to classify bleeding types like ulcers, angiodysplasia, or varices^10^. Supervised machine learning models, including convolutional neural networks (CNNs) and ensemble classifiers are trained on annotated datasets to recognize specific visual cues indicative of each bleeding type^11^. This dual approach of segmentation and classification not only enhances diagnostic precision but also facilitates prompt medical intervention and treatment planning tailored to the severity and nature of GI tract bleeding diseases^12^.

Existing approaches face several challenges in detecting, segmenting, and classifying gastrointestinal (GI) tract bleeding with technical rigor. The variability in bleeding appearances, influenced by factors like lighting conditions, tissue texture, and anatomical variation, complicates consistent detection across diverse patient populations^13^. Segmentation remains challenging due to the irregular shapes and sizes of bleeding areas, necessitating robust algorithms capable of handling complex and noisy WCE imagery. Moreover, the classification task requires distinguishing minute differences between bleeding types, which often manifest with overlapping visual features. This demands feature extraction techniques that effectively capture nuanced characteristics such as color distributions, texture patterns, and spatial context^14^. Also, the integration of real-time processing capabilities and the translation of algorithmic outputs into actionable clinical insights pose further technical hurdles^15^. Addressing these challenges requires advances in image processing methodologies and the integration of AI-driven decision support systems tailored to clinical workflows, ensuring reliable and efficient management of GI tract bleeding in medical practice. This research paper provides a novel method for the improved detection and classification of bleeding regions in GI tract wireless endoscopic images.

### Main contribution of this work

1. A novel Clip-BiRetinexNet is introduced that effectively enhances image contrast and color consistency while preserving fine details, such as edges and textures, which are crucial for accurately distinguishing between different types of bleeding, for an enhanced preprocessing of WCE images.
2. An improved segmentation with a novel Hough Canny-Frangi Enhanced DeepLabV3+ framework is proposed which combines Hough Canny edge detection and the Frangi Vesselness Filter with DeepLabV3+ thereby significantly improving the detection of non-linear and jagged edges, as well as the identification of fine vascular structures, to address the challenge of delineating irregular and complex borders of bleeding regions like ulcers and vascular lesions.
3. A Robust Classification with a novel ResNet-NaiveBayes Fusion is introduced for handling the complex patterns and textures of bleeding regions and ensuring high-level abstraction and probabilistic classification, contributing to the overall effectiveness and efficiency of the proposed system.

### The structure of the paper

The remaining content of this research manuscript is structured as follows: Section "Literature survey" provides an overview of existing literature, Section "Overall outline of the proposed methodology" explains the methodology and techniques proposed for this work, Section "Results and discussion" elaborates on the analytical findings related to performance and comparison to previous research, and Section "Conclusion" concludes the study.

## Literature survey

The detection and segmentation of gastrointestinal bleeding in wireless capsule endoscopy (WCE) images have been extensively explored through diverse deep learning and hybrid approaches. This section reviews key works in the field, highlighting their methodologies, significance, and limitations in the context of the challenges our proposed method aims to address.

### Review on deep learning-based segmentation and multi-pathology systems

Pedro et al.^16^ presented a multi-pathology system for WCE images using the Mask Improved RCNN (MI-RCNN), which employed a novel mask subnet strategy that significantly enhanced mask predictions of the previously high-performing state-of-the-art Mask-RCNN and PANet systems. For the first time, a unique training approach for Mask-RCNN and PANet-based systems was introduced, which was based on the second momentum. These methods were evaluated using the public database KID, and the included pathologies were inflammatory lesions, haemorrhage, polyps, and angioectasias. However, a limitation of this study was the reliance on the KID database, which may not encompass the full spectrum of pathologies encountered in WCE images.

Furqan Rustam et al.^17^ employed a deep neural network technique to categorize WCE bleeding images and proposed a model called BIR (Bleedy Image Recognizer), which integrated MobileNet with a specially designed convolutional neural network (CNN) model. The BIR model utilized MobileNet for initial-level calculations due to its lower computational resource requirements, and the resulting output was then processed further by the CNN. The model was trained and tested on a dataset of 1,650 WCE images, with its performance evaluated using Cohen’s kappa, F1 score, accuracy, precision, and recall metrics. However, this model do not fully capture the variability and complexity of bleeding images in WCE, potentially affecting the model’s generalizability to broader clinical scenarios. Additionally, the approach may have limited effectiveness in real-time applications due to the sequential nature of the processing stages.

Jain et al.^18^ proposed a deep convolutional neural network (CNN) based model called “WCENet” for anomaly identification and localization in WCE images. Initially, a straightforward and effective attention-based CNN was used to categorize an image into one of four groups: polyp, vascular, inflammatory, or normal. If the image was classified under one of the abnormal categories, it was processed in the second phase for anomaly localization. The segmentation of the anomalous region in the abnormal image was achieved by fusing Grad-CAM++ and a custom SegNet. The WCENet classifier achieved an accuracy of 98% and an area under the receiver operating characteristic of 99%. On the KID dataset, the WCENet segmentation model attained an average dice score of 56% and a frequency-weighted intersection over union of 81%. However, the relatively moderate dice score, indicates room for improvement in the precision of anomaly segmentation, particularly in complex and varied medical image scenarios.

### Review on feature fusion and advanced CNN architectures

Muhammad Attique Khan et al.^19^ demonstrated a completely automated approach based on the fusion and selection of deep learning features for the identification of stomach infections. This architecture supported a saliency-based ulcer identification approach by manually assigning ulcer images. Subsequently, transfer learning was employed to retrain the previously trained VGG16 deep learning model. Using an array-based method, the features of the retrained model were combined from two successive fully connected layers. Additionally, the top candidates were selected using the mean value-based fitness function and the metaheuristic technique known as PSO. Cubic SVM was eventually used to identify the chosen features. However, a limitation of this methodology was the reliance on manual assignment of ulcer images for the initial saliency-based identification, which could introduce bias and affect the overall automation and accuracy of the system in real-world applications.

Muhammad Sharif et al.^20^ utilized a novel method based on the combination of geometry and deep convolutional neural networks. Initially, a novel technique called contrast-enhanced color features was used to extract disease areas from WCE images. Geometric characteristics were then extracted from the segmented disease region. Subsequently, a unique feature fusion of VGG16 and VGG19 deep CNNs was performed based on the Euclidean Fisher Vector. To select the best features, the unique features were combined with geometric features and processed using a conditional entropy technique. Finally, the chosen attributes were classified using K-Nearest Neighbor. A confidentially gathered database containing 5,500 WCE images was employed to evaluate the proposed approach. However, the resource intensity of the combined feature extraction and fusion process, as well as the reliance on contrast-enhanced color features, which do not perform consistently across all types of WCE images, potentially affect the robustness and generalizability of the approach.

Samira Lafraxo et al.^21^ proposed an end-to-end 2D attention residual U-Net architecture (AttResU-Net) that further improved polyp and bleeding segmentation performance by integrating the attention mechanism and residual units into U-Net. In the downsampling and upsampling processes, AttResU-Net incorporated a series of attention units to minimize irrelevant regions in an input image while emphasizing prominent features. The residual block distributed information across layers, enabling the construction of a more complex neural network capable of addressing the vanishing gradient problem in each encoder. This approach reduced computational cost and enhanced channel interdependencies. However, the potential increase in model complexity due to the integration of attention units and residual blocks, which could lead to longer training times and increased demand for computational resources, possibly hindering real-time application and scalability.

### Review on optimization strategies and hybrid approaches

Goel et al.^22^ conducted a study that introduced a new approach for segmenting bleeding regions in WCE images through the implementation of an efficient deep neural network classifier algorithm. The design of the deep learning technique was enhanced by integrating feature extraction directly within the neural network, bypassing a separate feature extraction step to improve the productivity of the segmentation process. The study focused on optimizing two critical factors: the initial learning rate and the number of epochs. The model was tested with initial learning rate values of 0.009, 0.006, 0.003, 0.06, and 0.09, while varying the number of epochs from 1,000 to 10,000 iterations. These factors were deemed crucial in determining the performance of the segmentation algorithm. Segmentation accuracy was evaluated on 48 images from the KID dataset, resulting in a DICE rate of 69.99%. However, the achieved DICE rate indicated that there was room for improvement in the segmentation accuracy.

Using WCE pictures, Vrushali Raut et al.^5^ demonstrated a unique method for the segmentation and classification of GI tract illnesses. WCE pictures were first gathered from the KID Atlas collection and preprocessed using methods for contrast enhancement and filtering. After that, a modified U-Net architecture was used to execute lesion or abnormality segmentation, with parameters optimised using the Deer Hunting with Distance-based Solution Update (DH-DSU) algorithm. Then, a variety of techniques were used to extract features, such as the Grey Level Co-occurrence Matrix (GLCM), Scale-Invariant Feature Transform (SIFT), Local Binary Pattern (LBP), Histogram of Orientated Gradients (HOG), and Canny edge detection. The DH-DSU technique was utilised to control the optimisation of hidden neurones in an enhanced Deep Neural Network (DNN) after concatenating the collected features. Three GI tract conditions were included in the categorisation results: ulcer, polypoid, and inflammatory. However, asthis approach depends on the quality of the first segmentation phase, any errors in lesion or abnormality detection would affect the total diagnosis accuracy by extending to the classification step.

In order to accomplish bleeding segmentation in WCE pictures and to close the gap between Class Activation Maps (CAMs) and ground truths with the least number of annotations, Bai et al.^12^ presented a novel method called Discrepancy-basEd Active Learning (DEAL). In particular, precise model predictions and a small number of human labels were used to replace noisy CAMs with a novel discrepancy decoder model and a CAMPUS (CAM, Pseudo-label, and groUnd-truth Selection) criteria, which reduced manual work. A special training approach was used to provide standard, coarse, and fine predictions for the discrepancy decoder model. In addition, based on model and CAM divergence, the CAMPUS criteria were presented to forecast the differences between CAMs and ground facts. Still, the dependence on the CAMPUS criterion’s correctness presents a technical drawback since any error in determining the gaps between CAMs and ground truths might result in less-than-ideal segmentation and classification results.

### Review on ensemble models and recent advances

To forecast eight-class anomalies in digestive tract disorders, Chathurika et al.^23^ suggested using a global average pooling (GAP) layer after merging pre-trained DenseNet-201, ResNet-18, and VGG-16 CNN models as feature extractors. This would provide an ensemble of deep features as a single feature vector. Their results showed a noteworthy improvement over state-of-the-art methods, with a promising accuracy of 97%. In the context of transfer learning, the study examined the efficacy of well-known CNN architectures that have lately surfaced, such as DenseNet, ResNet, Xception, InceptionV3, InceptionResNetV2, and VGG. Furthermore, a method based on Singular Value Decomposition (SVD) feature extraction was presented to lower processing time and memory use without compromising the classification model’s accuracy. The possible difficulty of precisely segmenting and identifying bleeding sites is a technical problem of this methodology because, the ensemble approach’s complexity leads to overfitting or greater processing demands, which might affect performance in certain clinical circumstances.

Using endoscopic pictures, Marwa et al.^24^ demonstrated the Modified Salp Swarm Algorithm with Deep Learning-based Gastrointestinal Tract Disease Classification (MSSADL-GITDC). The primary objective of the MSSADL-GITDC approach was the analysis of WCE pictures for GIT categorisation. The MSSADL-GITDC methodology used the median filtering (MF) method for picture smoothing to accomplish this. A class attention layer (CAL) was added to the upgraded capsule network (CapsNet) model to provide a better model for feature extraction. To further maximise the performance of the upgraded CapsNet model, a hyperparameter tuning procedure based on the Modified Salp Swarm Algorithm (MSSA) was carried out. A deep belief network with an extreme learning machine (DBN-ELM) was used for GIT classification, and the DBN-ELM model was supervised fine-tuned by backpropagation. However, it would be difficult to precisely segment and classify bleeding areas due to the complexity brought about by the modified CapsNet and the MSSA-based tuning. This could also make it more difficult to generalise the model to other bleeding patterns.

Rathnamala et al.^25^ proposed an automated scheme for gastrointestinal bleeding detection in WCE images, in which input frames were first enhanced through distribution linearization and linear filtering and then segmented using an adaptive dense U-Net architecture. Feature points were extracted via an enhanced oriented FAST and rotated BRIEF (E-ORB) algorithm, and the classification of bleeding versus non-bleeding regions was performed using a “Multi-Enhanced Deep CapsNet” model. This approach was significant because it combined classical key-point features with deep learning classifiers, aiming to address both localization and classification of bleeding areas. However, its reliance on manual pre-processing and traditional feature extraction introduced complexity and potential brittleness to noise.

Sushma et al.^26^ developed “WCE-Bleed-Net,” a convolutional neural network augmented with an adaptive attention module, which was operated in an unsupervised pre-training setting via a convolutional autoencoder, followed by supervised classification with an Angular Contrastive (AC) loss to reduce intra-class variance and enhance inter-class separability. The approach was significant for addressing variability within bleeding classes, enhancing feature discrimination without requiring extensive labeled data. Nevertheless, its unsupervised reliance and AC-based discriminative training could limit its robustness across diverse lesion types and complex segmentation tasks.

Singh et al.^27^ presented “ColonNet,” a hybrid model integrating DenseNet-121 for feature extraction and U-Net for segmentation and classification of GI bleeding in WCE frames, evaluated using the Auto-WCBleedGen challenge dataset, where it achieved approximately 80% overall accuracy and ranked highest among 75 competing teams. The study highlighted the effectiveness of combining deep hierarchical features with segmentation models for bleeding detection. However, the moderate accuracy metric and absence of explicit segmentation quality indicators (e.g., Dice score) suggested limited precision in complex cases.

Sharmila et al.^28^ employed a combination of Minimum Spanning Tree (MST) analysis and Kudo’s pit pattern analysis to address the challenge of localizing and differentiating dyed regions from normal mucosal tissue in endoscopic images. MST was used to capture structural relationships of the mucosal surface by minimizing total edge weights while identifying regions of interest (ROIs). On these selected ROIs, Kudo’s pit pattern analysis was applied to evaluate morphological features, thereby improving the reliability of lesion detection and classification.

Sharmila et al.^29^ proposed a hybrid approach combining Minimum Spanning Tree (MST) analysis and Kudo’s pit pattern analysis for endoscopic image interpretation. MST was employed to capture the structural features of the mucosal surface and identify regions of interest by minimizing edge weights, while Kudo’s pit pattern analysis was applied on these regions to evaluate morphological characteristics. This integration enhanced the differentiation of dyed regions from normal mucosal tissue, thereby improving localization and classification performance in complex anatomical sites.

Table 1 shows the summary of reviewed techniques. Overall, existing systems for WCE bleeding detection face challenges in generalization due to limited or biased datasets, inability to fully capture variability and complexity of bleeding patterns, and reliance on manual or fixed feature extraction methods that may underperform under severe illumination changes. Many approaches suffer from moderate segmentation precision, particularly for complex or irregular lesion boundaries, and often propagate segmentation errors into classification. High computational complexity, long training times, and heavy models hinder real-time clinical applicability. Additionally, some methods lack explicit pixel-level segmentation or have architectures prone to overfitting, reducing robustness in diverse clinical scenarios. These limitations highlight the need for an integrated, efficient, and robust pipeline addressing both image quality enhancement and accurate lesion detection.

**Table 1:** Summary of survey on recent GI tract bleeding classification methods.

Author	Features used	Segmentation method	Classification method	Dataset
Pedro et al.^16^	CNN features	MI-RCNN, PANet with mask subnet	Integrated with segmentation	KID
Furqan Rustam et al.^17^	MobileNet + custom CNN features	None	MobileNet + CNN	1,650 WCE images
Jain et al.^18^	CNN features + attention maps	Grad-CAM++ + SegNet	WCENet CNN	KID
Muhammad Attique Khan et al.^19^	VGG16 transfer learning features + PSO selection	Saliency-based ulcer detection	Cubic SVM	Custom ulcer dataset
Muhammad Sharif et al.^20^	Geometric + CNN features (VGG16, VGG19)	Contrast-enhanced color feature extraction	KNN	5,500 WCE images
Samira Lafraxo et al.^21^	CNN attention features	AttResU-Net	Integrated	-
Goel et al.^22^	Integrated features in CNN	CNN segmentation	Same CNN	KID (48 images)
Vrushali Raut et al.^5^	GLCM, SIFT, LBP, HOG + CNN	Modified U-Net + DH-DSU	Enhanced DNN	KID Atlas
Bai et al.^12^	CAM-based features	Discrepancy decoder + CAMPUS	Integrated	-
Chathurika et al.^23^	DenseNet201 + ResNet18 + VGG16 ensemble features	None	GAP + SVD	WCE dataset
Marwa et al.^24^	CapsNet features + CAL	None	DBN-ELM	GI bleeding dataset
Rathnamala et al.^25^	Enhanced Oriented FAST and Rotated BRIEF (E-ORB) keypoints	Adaptive Dense U-Net	Multi-Enhanced Deep CapsNet	Public WCE image dataset (source not specified)
Sushma et al.^26^	CNN deep features with Adaptive Attention Module	Attention-augmented CNN feature maps (implicit segmentation via attention)	Angular Contrastive (AC) loss-based CNN classifier	WCE bleeding frames dataset (collected)
Singh et al.^27^	DenseNet-121 deep features	U-Net	DenseNet feature embeddings with integrated classification head	Auto-WCBleedGen Challenge Dataset

### Research motivation

The segmentation and classification of bleeding regions in gastrointestinal (GI) tract endoscopic images, particularly within the context of wireless capsule endoscopy (WCE), hold primary importance. The accurate segmentation and classification of bleeding types in WCE images are crucial for timely and precise diagnosis of GI tract conditions. Despite advancements in endoscopic technology and image processing, existing methods fall short of effectively addressing the challenges posed by different bleeding types. Erosions and ulcers, for instance, require precise detection of their borders and depth, which can be obscured by mucosal folds or debris. Also, fine vascular lesions demand differentiation from normal vascular patterns and other anomalies.

Active bleeding has a bright red appearance, while fresh clots can be dark red or maroon, and old stains appear dark brown or black. Existing feature extraction algorithms do not well capture the fine details and differences present in the bleeding images because of ineffective colour differentiation and texture pattern recognition capabilities. Most of them do not effectively combine colour and texture information to enhance the discriminative power required for accurate bleeding type classification thereby missing to capture fine-scale textures that differentiate between surface characteristics of active bleeding (smooth, possibly glistening) versus coagulated blood (blood clot i.e., clumpy or irregular textures).

Moreover, some specific bleeding types, such as ulcers and vascular lesions often have irregular and complex borders, making it difficult for the existing segmentation and classification approaches to delineate them accurately because of the lack of effective border detection and are less effective in identifying vascular lesion anomalies. Existing methods typically rely on edge detection algorithms that struggle with the non-linear and jagged edges characteristic of these lesions. Also, vascular lesions present as faint and delicate patterns that are easily missed by algorithms not specifically designed to detect fine vascular structures. Hence, this research presents a novel approach for accurately classifying bleeding regions in the GI tract from WCE images.

## Overall outline of the proposed methodology

**Fig. 1:**
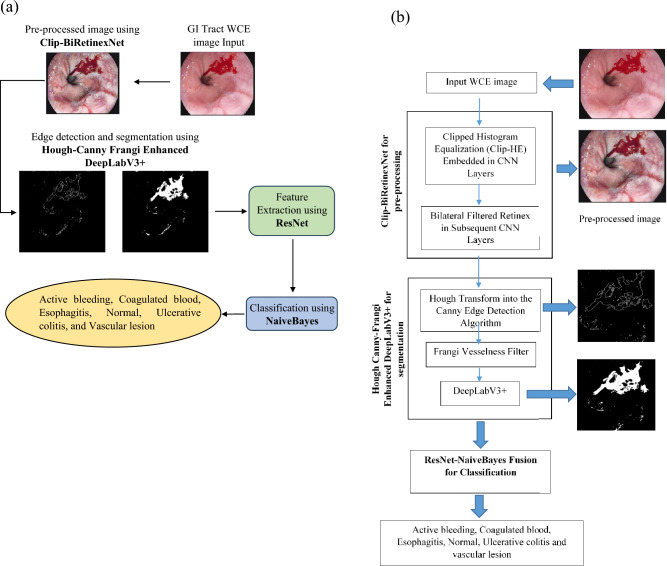
(**a**) Overall Process flow of the proposed model. (**b**) Architecture and components of the proposed GI tract disease detection model.

To address the challenges in segmenting and classifying bleeding types in WCE images, a novel Multi-Stage Convo-Enhanced Retinex Canny DeepLabV3+ FusionNet is proposed. This methodology incorporates Clip-BiRetinexNet for pre-processing, Hough Canny-Frangi Enhanced DeepLabV3+ for segmentation and ResNet-NaiveBayes Fusion for Classification. The overall process flow of the proposed architecture is shown in Figure 1(a).

It begins with the Clip-BiRetinexNet for pre-processing, where WCE images are enhanced using a combination of Clipped Histogram Equalization and Bilateral Filtered Retinex within a CNN. This step improves image contrast, reduces noise, and corrects for varying lighting conditions, enabling better preservation and enhancement of critical features like edges, textures, and colours. This preprocessing makes features more consistent and discernible, setting the stage for effective classification. In the segmentation phase, a DeepLabV3+ network is augmented with Hough Canny edge detection and the Frangi Vesselness Filter. The Hough Canny method enhances the connection of disjoint edge segments, allowing for more continuous and meaningful edge detection in regions with irregular bleeding patterns. The Frangi Vesselness Filter, specifically designed for vascular lesion detection, highlights blood vessels by enhancing the contrast of vascular patterns, making them easier to detect and differentiate from normal tissue. These enhancements make the subsequent segmentation by DeepLabV3+ highly accurate, even for complex borders typical of bleeding lesions. The detailed architecture and the components of the proposed model are presented in Figure 1(b).

Finally, in the classification stage, ResNet-NaiveBayes Fusion is introduced in which ResNet is used for feature extraction from the segmented regions. Its deep learning architecture captures high-level patterns specific to bleeding types. These extracted features are then fed into a Naive Bayes classifier, which provides efficient and interpretable classification of the bleeding types with classes such as Active bleeding, Coagulated blood, Esophagitis, Normal, Ulcerative colitis and Vascular lesion. The combination of ResNet’s feature extraction capabilities and the simplicity and speed of Naive Bayes ensures a powerful yet computationally efficient framework that is suitable for real-time medical diagnostics. The working of each component of the proposed segmentation and classification method is provided in the upcoming subsections.

### Clip-BiRetinexNet for pre-processing

Although modern CNN architectures can learn invariance to moderate lighting variations, WCE imaging presents extreme illumination inconsistencies caused by varying distances of the capsule light source to mucosal surfaces, strong specular reflections and shadows and rapid changes in tissue colour due to peristalsis and bile staining. These variations cause intra-class variability and inter-class confusion, particularly in early layers of the CNN. Hence, the proposed pre-processing stage is designed to systematically enhance the input Wireless Capsule Endoscopy images, which typically suffer from variable lighting conditions, low contrast, and noise. This process is critical for ensuring that important features, such as bleeding regions, are effectively highlighted, thus enabling more accurate downstream segmentation and classification. In the novel Clip-BiRetinexNet model, the CNN architecture is directly embedded with Clipped Histogram Equalization (Clip-HE) and Bilateral Filtered Retinex within its subsequent processing layers, ensuring that the network learns both from enhanced contrast and corrected lighting conditions during the feature extraction process. Initially, the raw WCE image is input into the network through the first CNN layer. Unlike conventional CNNs, where feature extraction begins without any preprocessing, this novel architecture integrates Clip-HE and Bilateral Filtered Retinex directly into the network’s processing flow. This design enables the network to automatically enhance contrast and correct lighting as part of the convolutional operations.

The CNN architecture used in this Clip-BiRetinexNet comprises 8 convolutional layers, organized into two primary processing stages: Stage 1 for Clipped Histogram Equalization (Clip-HE) and Stage 2 for Bilateral Filtered Retinex (BFR). Each convolutional layer uses a 3x3 kernel with a stride of 1 and ReLU activation to effectively capture spatial and edge details. In Stage 1, the first two convolutional layers integrate Clip-HE, where pixel intensity histograms are dynamically calculated and clipped based on a predefined threshold. Stage 2 introduces BFR within the next three convolutional layers, refining image details. The final three convolutional layers aggregate hierarchical features to further enhance the image. The output from this process is a pre-processed image characterized by improved contrast, sharper edges, and reduced noise, which is optimized for subsequent tasks such as segmentation and classification.

#### Clipped histogram equalization (Clip-HE) embedded in CNN layers

In the early convolutional layers, the Clip-HE process is embedded. The pixel intensity distribution is analyzed dynamically within these layers, and based on the intensity histogram, clipping is applied before histogram equalization. This ensures that the contrast is uniformly enhanced across the feature maps generated by the CNN, avoiding the risk of noise amplification. The subsequent layers then refine the contrast of the feature maps, providing balanced enhancement that is crucial for retaining fine diagnostic details, such as subtle bleeding regions, without overwhelming the network with noise.

Mathematically, Clip-HE involves modifying the cumulative distribution function (CDF) of the image’s intensity histogram. Let $I(x,y,c)$ represent the pixel intensity at the position $(x,y)$ in channel $"c"$ of the input WCE colour image; where, $\text{c}\in \{\text{R},\text{G},\text{B}\}$. For each colour channel, the histogram is calculated separately. The histogram ${H}_{c}(i)$ for channel $"c"$ is defined as given in equation (1)

1 $${H}_{c}\left(i\right)= \sum_{x=1}^{M}\sum_{y=1}^{N}\delta \left[I\left(x,y,c\right)-i\right]$$

Where, $\delta$ is the Dirac delta function, $M\times N$ is the size of the image, and $i$ ranges from 0 to 255 for the intensity values. The histogram ${H}_{c}(i)$ represents the frequency distribution of intensity values in each channel, which is then used to identify regions with low contrast or uneven intensity distribution across the color components of the WCE image.

The next step involves clipping of histogram bins for each channel in which a predefined clipping threshold ​${T}_{clip,c}$ is set for each colour channel c. This threshold is determined as a specific intensity threshold for each channel. The clipping process limits the maximum value of any bin in the histogram to ${T}_{clip,c}$. This clipping process for ach channel is mathematically expressed as follows in equation (2)

2 $${H}_{clipped,c}\left(i\right)=\text{min}\left[{H}_{c}\left(i\right), {T}_{clip,c}\right]$$

If ${H}_{c}\left(i\right)>{T}_{clip,c}$, the bin is clipped to ${T}_{clip,c}$, ensuring that pixel values concentrated in certain intensity ranges (usually due to noise or irrelevant details) do not dominate the contrast enhancement process in each colour channel. After clipping, the excess pixel counts from high-frequency bins in each channel $c$ are redistributed uniformly across all intensity levels in that channel. The total number of excess pixels after clipping for channel $c$ is represented as ${E}_{c}$ and given is equation (3)

3 $${E}_{c}= \sum_{i=0}^{255}\text{max}[0, {H}_{c}\left(i\right)- {T}_{clip,c}]$$

During the histogram clipping process, certain intensity bins may exceed the predefined threshold ${T}_{clip,c}$ due to concentrated pixel values (often noise or irrelevant details). If these excess pixels are simply discarded, the histogram would become unbalanced, leading to a loss of brightness consistency and potential artifacts in the enhanced image. To prevent this, the excess pixel counts ${E}_{c}$ are redistributed uniformly across all intensity levels within each color channel as expressed in equation (4)

4 $${H}_{final,c}\left(i\right)= {H}_{clipped,c}\left(i\right)+\frac{{E}_{c}}{256}$$

In equation (4), the division by 256 is performed because the redistribution process considers the full range of intensity levels, which includes all 256 possible values from 0 to 255 (including 0). Each intensity level is treated as a valid bin for pixel redistribution, and the division by 256 ensures that the excess ${E}_{c}$ is distributed equally across all bins. This approach ensures a uniform balance across the entire intensity spectrum, maintaining consistency in brightness and contrast across the image. Using 255 instead of 256 would result in an uneven redistribution, with some bins potentially being under-enhanced, thereby affecting the final image quality.

Once the histogram has been clipped and the excess redistributed, Histogram Equalization is applied to each colour channel using the modified histogram. The Cumulative Distribution Function (CDF) for each channel $c$ is calculated as given in equation (5)

5 $${C}_{c}\left(i\right)= \frac{1}{M \times N}\sum_{j=0}^{i}{H}_{final,c}\left(i\right)$$

The CDF normalizes the histogram, converting it into a probability distribution function for each channel. The original pixel intensities $I(x,y,c)$ are remapped to new intensities $I{\prime}(x,y,c)$ using the CDF for each channel and is expressed in the following equation (6)

6 $${I}{\prime}\left(x,y,c\right)=255 \times {C}_{c}\left[I\left(x,y,c\right)\right]$$

This remapping ensures that the intensity range in each colour channel is fully utilized, enhancing the contrast of the colour WCE image while maintaining colour balance. The difference from traditional HE is that by using the clipped and redistributed histograms, the over-enhancement of noise and irrelevant details is prevented leading to a more controlled contrast enhancement across all colour channels.

By embedding Clip-HE into the early layers, the network can better discriminate between areas of low contrast and high contrast, allowing the CNN to amplify significant regions (e.g., lesions) while suppressing irrelevant features. This step ensures that the CNN is trained on feature maps where diagnostic regions are more prominent and noise is minimized, improving the robustness of the learning process. Following this, the processed image is subjected to Bilateral Filtered Retinex to better preserve edges while reducing noise and enhancing details and edges more effectively which is explained below.

#### Bilateral filtered retinex in subsequent CNN layers

The Bilateral Filtered Retinex (BFR) process is integrated within the subsequent CNN layers following the contrast enhancement performed by Clip-HE. In this stage, the network simulates human-like colour perception by smoothing illumination while preserving critical edges. The bilateral filter operates in tandem with the convolutional filters to maintain edge integrity while reducing noise, ensuring that anatomical structures such as blood vessels and lesion boundaries remain distinct. Traditional filtering techniques, such as Gaussian filters, tend to blur the entire image, including edges, which can result in the loss of critical diagnostic details. The Bilateral Filtered Retinex, however, accounts for both spatial and intensity differences when smoothing the image. This allows it to selectively reduce noise in homogenous regions (such as tissue surfaces) while preserving high-contrast boundaries (such as the edges of lesions or bleeding sites).

After the Clipped Histogram Equalization stage, the enhanced image ${I}{\prime}\left(x,y,c\right)$, with improved contrast for each colour channel $c\in \{R,G,B\}$ is processed using Bilateral Filtered Retinex to address the challenges posed by varying illumination and noise. Initially, the Retinex process within these CNN layers works by separating the illumination and reflectance components of the image. Here in this process, $L(x,y,c)$ denotes the illumination component which represents the varying lighting conditions across the image due to the uneven lighting conditions inside the body during endoscopy; and $R(x,y,c)$ denotes the reflectance component representing the intrinsic colour properties of the scene, which remains consistent under different lighting conditions. Mathematically, this decomposition is expressed as shown in equation (7);

7 $${I}{\prime}\left(x,y,c\right)=L\left(x,y,c\right)\cdot R\left(x,y,c\right)$$

Where, $I{\prime}(x,y,c)$ is the intensity of the pixel at position $(x,y)$ for channel $c$, which has already been processed by Clip-HE. But, the objective of the Retinex model is to estimate the reflectance $R(x,y,c)$ by isolating the illumination $L(x,y,c)$. To achieve the decomposition, bilateral filtering is applied to smooth the illumination component while preserving the edges of the reflectance component. Bilateral filtering is a non-linear filter that preserves edges by taking into account both spatial proximity and pixel intensity differences. For each colour channel $c$, the bilateral filter is applied to $I{\prime}(x,y,c)$ to estimate the illumination component $L(x,y,c)$. The bilateral filter output is given by equation (8)

8 $$L\left(x,y,c\right)=\frac{1}{W\left(x,y\right)}\sum_{\left({x}{\prime},{y}{\prime}\right)\in {P}_{n}}{I}{\prime}\left({x}{\prime},{y}{\prime},c\right)\cdot {f}_{s}\left(\left|\left|\left(x,y\right)-\left({x}{\prime},{y}{\prime}\right)\right|\right|\right)\cdot {f}_{r}\left(\left|{I}{\prime}\left(x,y,c\right)-{I}{\prime}\left({x}{\prime},{y}{\prime},c\right)\right|\right)$$

Where, ${P}_{n}$ is the neighborhood of pixel $(x,y)$; $W(x,y)$ is a normalization factor; ${f}_{s}(||(x,y)-(x{\prime},y{\prime})||)$ is the spatial Gaussian kernel, controlling the influence of neighbouring pixels based on their distance from the central pixel; ${f}_{r}(\left|{I}{\prime}\left(x,y,c\right)-{I}{\prime}\left({x}{\prime},{y}{\prime},c\right)\right|)$ is the range Gaussian kernel, controlling the influence of neighboring pixels based on their intensity similarity to the central pixel.

This bilateral filter effectively smooths the illumination while preserving the sharpness of edges. Once the illumination $L(x,y,c)$ is estimated for each color channel $c$, the reflectance $R(x,y,c)$ is computed by dividing the processed image $I{\prime}(x,y,c)$ by the estimated illumination as given by equation (9)

9 $$R\left(x,y,c\right)=\frac{{I}{\prime}\left(x,y,c\right)}{L\left(x,y,c\right)+\varepsilon }$$

Where,$\varepsilon$ is a small constant added to avoid division by zero. This operation yields the reflectance component, which represents the corrected colour and texture information, making it invariant to the illumination variations in the original image. The reflectance component $R(x,y,c)$ is the final output of the Bilateral Filtered Retinex process. This enhanced image retains the edge details (such as blood vessels and lesion boundaries) while reducing the effects of non-uniform lighting. The enhanced colour constancy and colour differentiation provided by Bilateral Filtered Retinex enable the network to distinguish between different types of tissues, highlighting the contrast between healthy and abnormal areas.

**Algorithm 1** Clip-BiRetinexNet Pre-processing for WCE Images
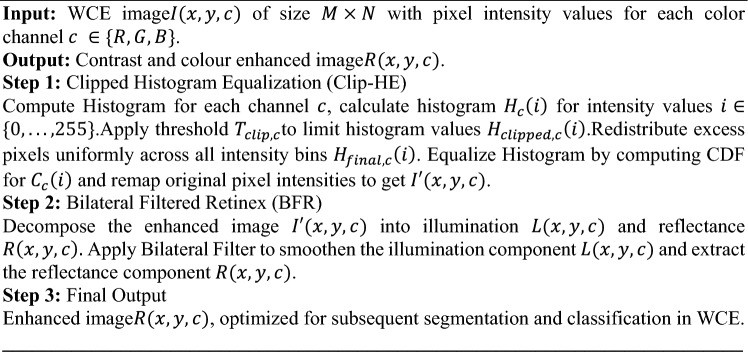


The algorithm for the proposed Clip-BiRetinexNet for pre-processing is provided in Algorithm 1. By integrating the Clipped Histogram Equalization and Bilateral Filtered Retinex methods within a CNN, this approach learns hierarchical features from data, which is crucial in identifying fine details such as edges, textures, and colours that need to be preserved and enhanced and captures high-level, abstract features that are more discriminative for the classification of different bleeding types. Following this pre-processing, the proposed approach involves segmentation using a novel deep learning approach which is discussed in the upcoming section.

### Hough canny-frangi enhanced DeepLabV3+ for segmentation

DeepLabV3+ is selected for the segmentation stage because it effectively handles irregular lesion boundaries through the use of atrous convolutions and multi-scale context aggregation via Atrous Spatial Pyramid Pooling (ASPP). This architecture enables accurate detection of bleeding regions even when they appear fragmented, small, or have low contrast with surrounding tissue. In this stage, the pre-processed image, enriched with enhanced contrast and corrected colour consistency, is passed through a DeepLabV3+ augmented with Hough Canny edge detection and the Frangi Vesselness Filter for segmentation. This approach introduces a robust segmentation framework designed specifically to handle complex bleeding regions and vascular lesions in Wireless Capsule Endoscopy images. The pre-processed image $R(x,y,c)$ which represents an enhanced image with improved contrast, better lighting consistency, and edge details obtained from the Clip-BiRetinexNet is the input to this segmentation stage. Initially, the Hough Canny edge detection algorithm is integrated into DeepLabV3+ to enhance border detection. This method integrates Hough Transform into the Canny Edge Detection Algorithm to enhance the edge linking process to better connect disjoint edge segments and help in forming more continuous and meaningful edges particularly those characteristic of bleeding regions. Hence, this approach is adept at handling the non-linear and jagged edges characteristic of bleeding regions.

The Canny Edge Detection Algorithm is the starting point, where the image is first smoothed using a Gaussian filter $G\left(x,y,c\right)$ to reduce noise while preserving the essential structural details. This reduces the effect of noise that could lead to false edge detection. The smoothed image is mathematically represented as follows in equation (10)

10 $${R}_{s}\left(x,y,c\right)= G\left(x,y,c\right). R\left(x,y,c\right)$$

Then, the intensity gradient is calculated to detect prominent edges across the image by generating an initial edge map that identifies key transitions in pixel intensity. The intensity gradient $\nabla I(x,y,c)$ represents the change in intensity across the spatial coordinates x and y for a specific color channel $c$. It is typically represented as a vector of the partial derivatives in the x- and y-directions as given by equation (11)

11 $$\left|\nabla I\left(x,y,c\right)\right|= \sqrt{{\left(\frac{\partial {R}_{s}(\text{x},\text{y},\text{c}) }{\partial \text{x}}\right)}^{2}+{\left(\frac{\partial {R}_{s}\left(\text{x},\text{y},\text{c}\right) }{\partial \text{y}}\right)}^{2}}$$

Where, $\frac{\partial {R}_{s}(\text{x},\text{y},\text{c}) }{\partial \text{x}}$ is the horizontal gradient and $\frac{\partial {R}_{s}\left(\text{x},\text{y},\text{c}\right) }{\partial \text{y}}$ is the vertical gradient.

After detecting the initial edge segments using this intensity gradient from Canny edge detection, the Hough Transform is integrated into the Canny Edge Detection Algorithm to enhance the connectivity of disjointed edge segments. This is particularly useful in detecting non-linear, jagged edges in bleeding regions.

Here, the edge points are mapped into a parametric space where lines are detected more robustly. For each edge point $({x}_{i},{y}_{i})$, a line is parameterized by the following equation (12)

12 $$\rho = {x}_{i} cos\theta + {y}_{i} sin\theta$$

Where, $\rho$ is the distance from the origin, and $\theta$ is the angle of the normal to the line.The algorithm ensures that broken or non-linear bleeding region borders in ${R}_{s}(x,y,c)$ are more coherent by identifying continuous geometric structures in this transformed parametric space. By combining Canny edge detection with the Hough Transform and voting in the Hough space, the continuous lines are detected and disjoint segments of edges are connected more effectively. Hence, the enhanced edge map is obtained by combining the connected edges, ensuring that previously disjoint segments are now continuous.

The resultant image with edge enhancement after applying Hough Transform is represented as given in equation (13)

13 $$E(x,y,c)=Hough Transform(\nabla I(x,y,c))$$

This results in smoother and continuous boundary representations for complex lesions and irregular bleeding regions. These refined edges are then fed back into the network to guide the feature extraction and segmentation process.

Subsequently, the Frangi Vesselness Filter is employed for vascular lesion detection, which targets the enhancement of tubular and vascular structures within the image. This filter operates by computing eigen-values of the Hessian matrix at each pixel, which measure the curvature of intensity levels in different directions. The eigenvalues capture the curvature along different axes, allowing the filter to highlight structures that resemble vessels.

Frangi Vesselness Filtering is applied to $E(x,y,c)$ to enhance vascular structures and tubular patterns in the image. The Frangi Vesselness Filter operates on the eigenvalues ${{\varvec{\lambda}}}_{1}$ and ${{\varvec{\lambda}}}_{2}$ of the Hessian matrix ${\varvec{H}}$ at each pixel, which is shown in equation (14)

14 $$H= \left[\begin{array}{cc}\frac{{\partial }^{2}E}{{\partial x}^{2}}& \frac{{\partial }^{2}E}{\partial x\partial y}\\ \frac{{\partial }^{2}E}{\partial y\partial x}& \frac{{\partial }^{2}E}{{\partial y}^{2}}\end{array}\right]$$

Then, the Vesselness Function ${\varvec{V}}({\varvec{x}},{\varvec{y}},{\varvec{c}})$ amplifies the intensity of tubular shapes, making blood vessels and other vascular patterns more prominent. This Vesselness function is defined to emphasize tubular structures and is mathematically expressed as follows in equation (15);

15 $$V\left(x,y,c\right)=\text{exp}\left(-\frac{{R}_{b}^{2}}{{2\upbeta }^{2}}\right).\left(1-exp\left(-\frac{{S}^{2}}{{2\text{c}}^{2}}\right)\right)$$

Where, ${R}_{b}=\frac{\left|{{\varvec{\lambda}}}_{2}\right|}{\left|{{\varvec{\lambda}}}_{1}\right|}$, $S= \sqrt{{{{\varvec{\lambda}}}_{1}}^{2}+{{{\varvec{\lambda}}}_{1}}^{2}}$ and $\beta$ is a parameter that controls the sensitivity of the filter to the roundness of the structure, which is captured by the ratio of the eigenvalues ${R}_{b}.$ This $\beta$ is used to tune how much the filter suppresses structures that do not resemble tubular shapes. A lower $\beta$ value will increase the sensitivity to elongated, tube-like structures, while a higher $\beta$ value allows more variation in shape. This parameter helps distinguish tubular structures (like blood vessels) from other features in the image. Essentially, $\beta$ influences the weight given to the roundness term ${R}_{b}$ in the Vesselness Function, helping to discriminate between vessel-like and non-vessel-like structures. This process enhances the contrast between blood vessels and surrounding tissues, making vascular patterns more pronounced and easier to identify and aiding in the accurate identification and differentiation of vascular lesions from normal tissue.

The output image at this stage is the result of applying the Frangi Vesselness Function to the edge-enhanced image $E(x,y,c)$. Thisoutput image, denoted by ${I}_{Output}(x,y,c)$, is given by the following equation (16)

16 $${I}_{Output}\left(x,y,c\right)=V \left(x,y,c\right).E\left(x,y,c\right)$$

This represents the combination of the edge-enhanced image with the Vesselness function, highlighting both the vascular lesions and the bleeding regions with enhanced edges and tubular structures.

Finally, this output image ${I}_{Output}\left(x,y,c\right)$, enriched with detailed edge and vascular information, is passed through the final processing layer of DeepLabV3+ to segment the regions accurately.DeepLabV3+ utilizes atrous (dilated) convolution to adjust the receptive field of convolutional filters dynamically. The addition of Atrous Spatial Pyramid Pooling (ASPP) in DeepLabV3+ further enhances its capability to process features at different scales simultaneously, which is essential for correctly identifying both large and small lesions with varying texture and border characteristics. This is useful for segmenting regions with irregular and complex borders, such as ulcers and vascular lesions.

The Atrous Spatial Pyramid Pooling in DeepLabV3+ applies atrous convolution on the image ${I}_{Output}\left(x,y,c\right)$ to generate an output feature maprepresented by equation (17)

17 $${F}_{Output}\left(x,y,c\right)= \sum_{i=1}^{n}{W}_{i}. R[\left(x+{r}_{i}\right), \left(y+{r}_{i}\right),c)]$$

Where, ​ ${W}_{i}$ are the learned convolutional weights, and ${r}_{i}$ represents the dilation rate of the filter, which controls the receptive field size. The final output is a segmented mask that accurately delineates pathological regions with clear boundaries, informed by the edge continuity and vascular structure enhancements provided earlier in the process. The final segmentation mask $M(x,y,c)$ is obtained by applying a softmax function to the output of the final layer in DeepLabV3+ and is shown in equation (18)

18 $$M\left(x,y,c\right)=softmax \left[{F}_{Output}\left(x,y,c\right)\right]$$

This generates a pixel-wise probability distribution over different classes and the class with the highest probability is assigned to each pixel.

**Algorithm 2** Hough Canny-Frangi Enhanced DeepLabV3+ Algorithm
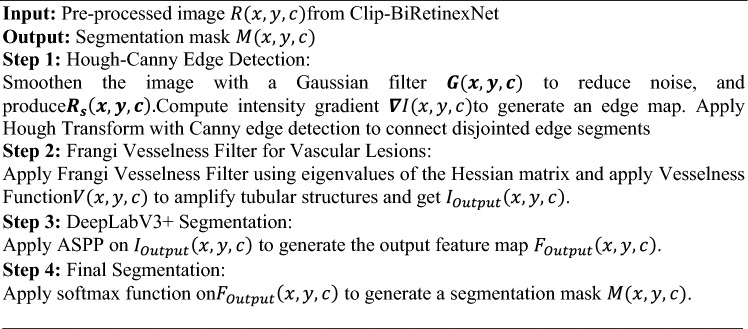


The algorithm for Hough Canny-Frangi Enhanced DeepLabV3+ is presented in Algorithm 2. By integrating edge detection, vascular enhancement, and multi-scale feature extraction, the Hough Canny-Frangi Enhanced DeepLabV3+ model achieves superior segmentation performance, particularly for intricate and irregular regions such as bleeding zones and vascular lesions. The combination of these techniques results in higher segmentation accuracy and more reliable identification of bleeding conditions within the GI tract. The technical aspects of the proposed novel classification stage is discussed in the next section.

### ResNet-naivebayes fusion for classification

**Fig. 2:**
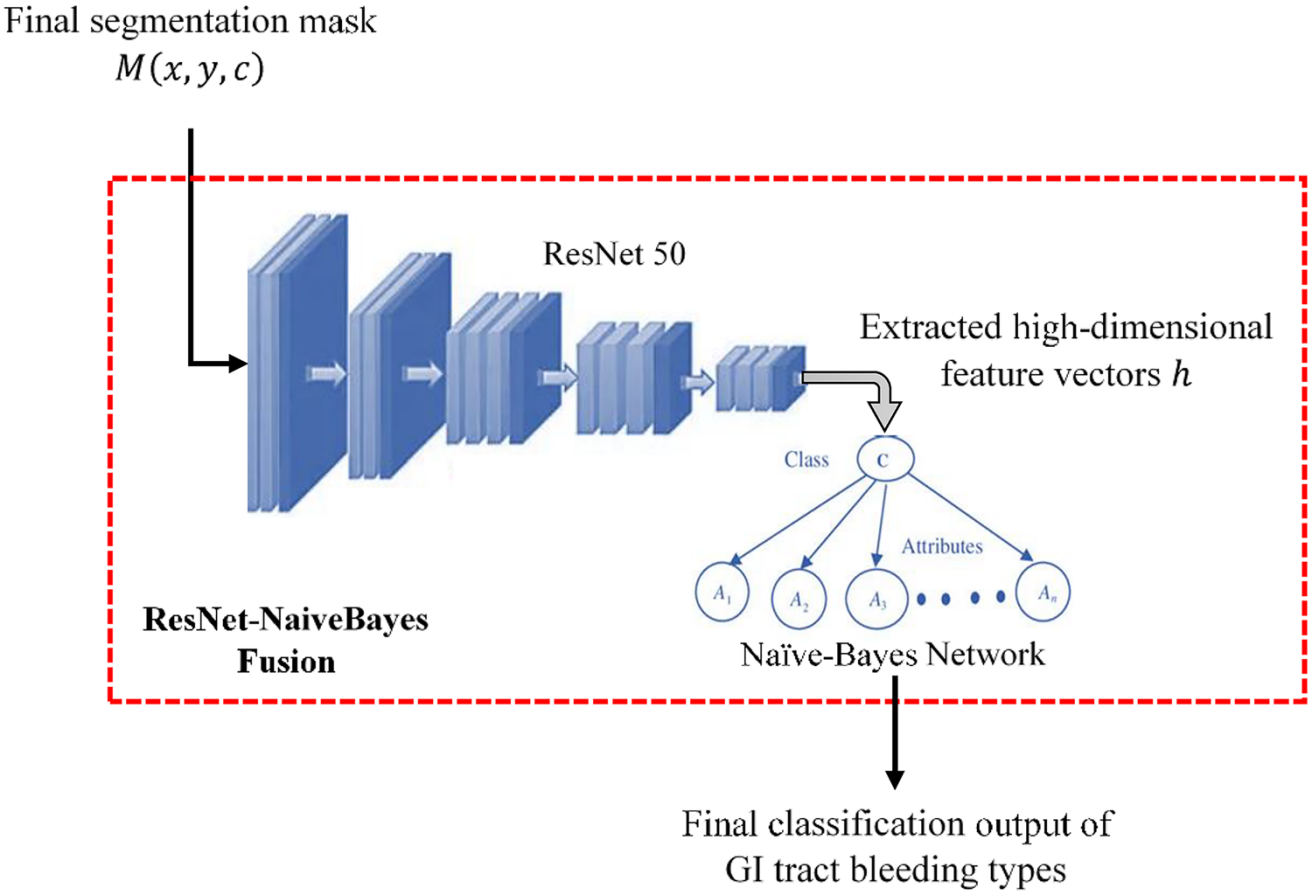
ResNet-naivebayes fusion architecture.

For classification, the ResNet–Naive Bayes fusion approach is adopted to combine the representational power of deep residual networks with the computational efficiency of a probabilistic classifier. ResNet serves as a robust feature extractor, producing discriminative embeddings, while the Naive Bayes classifier provides lightweight and fast classification. This combination ensures high accuracy while keeping the model efficient, making it well-suited for real-time clinical use where processing speed is critical. In the final stage of the proposed methodology for classifying bleeding types in WCE images, a combination of ResNet50 for feature extraction and a Naive Bayes classifier termed as ResNet-NaiveBayes Fusion for classification is employed. The architecture of ResNet-NaiveBayes Fusion is depicted in Figure 2.

This approach leverages the deep learning capabilities of ResNet50 to capture complex patterns and textures from the segmented regions provided by the Hough Canny-Frangi Enhanced DeepLabV3+ segmentation stage. ResNet, a highly successful convolutional neural network architecture, is utilized for its ability to learn high-level, abstract features through its deep layers, which are essential for differentiating between various types of bleeding. By fine-tuning ResNet50 on the specific dataset, it is ensured that the extracted features are tailored to the characteristics of bleeding regions in WCE images.

The segmented regions from the final segmentation mask $M\left(x,y,c\right)$ are forwarded through the ResNet50 layers to capture low-level and high-level patterns. The ResNet50 model consists of several convolutional blocks (Residual Blocks), which are mathematically represented as given in equation (19)

19 $${F}_{L+1}\left(x\right)={F}_{L}\left(x\right)+{R}_{f}\left({F}_{L}\left(x\right)\right)$$

Where, ${F}_{L}\left(x\right)$ is the output of the $L$-th layer and ${R}_{f}$ is the residual function consisting of convolutional layers followed by batch normalization and ReLU activation. The residual connections help in the training of very deep networks by preserving information from previous layers.The output of ResNet is a high-dimensional feature vector $h$, which encodes the patterns, textures, and specific bleeding characteristics of the input image and is represented as: $h= {f}_{ResNet}\left(M\left(x,y,c\right)\right)$; where, ${f}_{ResNet}$ represents the function learned by the ResNet50 architecture.

Following feature extraction, the high-dimensional feature vectors $h$ produced by ResNet are fed into a Naive Bayes classifier for the final classification task. The Naive Bayes classifier is chosen for its simplicity, efficiency, and probabilistic nature, making it well suited for handling the classification task. In this fusion process, the output feature vector $h$ is directly fed into a Naive Bayes classifier. The Naive Bayes classifier treats each feature in $h$​ as a separate input dimension and assumes that these features are conditionally independent given the class label and then computes the posterior probability for each bleeding class (Normal, Active bleeding, Coagulated blood, Vascular lesion, Ulcerative colitis, and Esophagitis).

The posterior probability for class ${C}_{i}$ given the feature vector $h$ is computed as given by equation (20)

20 $$P\left({C}_{i}|h\right)= \frac{P\left({C}_{i}\right).P\left({h|C}_{i}\right)}{P\left(h\right)}$$

Where, $P\left({C}_{i}\right)$ is the prior probability of class ${C}_{i}$,$P\left({h|C}_{i}\right)$ is the likelihood of observing the feature vector $h$ given that the class is ${C}_{i}$, and $P(h)$ is the evidence or marginal probability of the feature vector $h$. Then, in this Naive Bayes, the conditional independence assumption is made, where each feature ${h}_{j}$​ is conditionally independent of the others given the class label ${C}_{i}$. Thus, the likelihood $P\left({h|C}_{i}\right)$ is simplified as the product of the individual probabilities for each feature as given by equation (21)

21 $$P\left({h|C}_{i}\right)= \prod_{j=1}^{n}P\left({h}_{j}|{C}_{i}\right)$$

Where, $P\left({h}_{j}|{C}_{i}\right)$ is the probability of $j$ th feature given class ${C}_{i}$.

For the final classification decision, this Naive Bayes classifier assigns the class label $\widehat{C}$ to the input image based on the maximum posterior probability which is mathematically expressed by equation (22)

22 $$\widehat{C}=\text{arg}\underset{{C}_{i}}{\text{max}}P\left({h|C}_{i}\right)$$

This classifier selects the class ${C}_{i}$ that maximizes the posterior probability, effectively classifying the image into one of the bleeding types: Normal, Active bleeding, Coagulated blood, Vascular lesion, Ulcerative colitis, or Esophagitis. While this independence assumption is a simplification, it works effectively in combination with the highly discriminative feature representations generated by ResNet50.

**Algorithm 3** ResNet-NaiveBayes fusion for classification
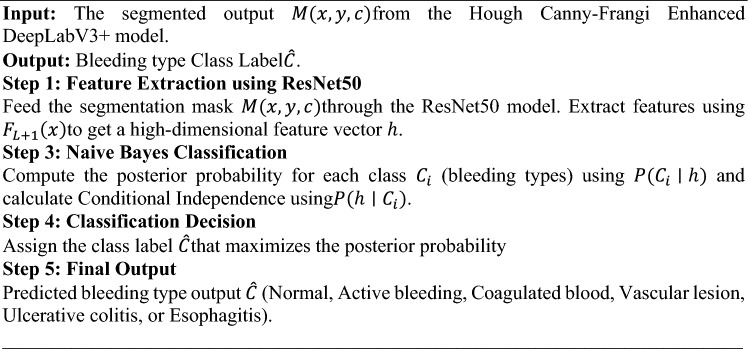


The proposed ResNet-NaiveBayes Fusion algorithm for classification is shown in Algorithm 3. This integrated approach not only enhances the accuracy of detecting and classifying different bleeding types but also ensures that the entire process remains computationally feasible and effective for real-time medical diagnostics.

### Justification of architectural decisions and hyperparameters

The proposed architecture was designed to address the specific challenges of gastrointestinal tract image classification, particularly in Wireless Capsule Endoscopy (WCE), where variable illumination, low contrast, presence of specular highlights, and subtle lesion boundaries hinder reliable automated detection.

1. ***Preprocessing (Clip-BiRetinexNet):*** Selected to correct severe lighting variations and enhance local contrast without over-amplifying noise. The clipping threshold Tclip,cT_{clip,c}Tclip,c​ was empirically tuned to 2% of the histogram peak per channel, as this setting provided the best trade-off between preserving fine details and avoiding saturation artifacts.
2. ***Segmentation (Hough–Canny–Frangi + DeepLabV3+):*** DeepLabV3+ was chosen for its atrous convolution and multi-scale context aggregation (ASPP), which are effective for irregular lesion shapes common in GI tract images. The hybrid pre-segmentation (Hough–Canny–Frangi) was integrated to enhance vascular and bleeding patterns before feeding to DeepLabV3+, improving boundary precision for vascular lesions and small hemorrhages.
3. ***Classification (ResNet–Naïve Bayes Fusion):*** ResNet50 was selected as the feature extractor for its residual connections that mitigate vanishing gradients and its proven robustness in medical image analysis. Naïve Bayes was preferred over other lightweight classifiers (SVM, kNN) due to its low computational cost (<0.04 s/frame) and superior classification accuracy on extracted features (99.2% vs. 98.6% for SVM, 98.1% for kNN).
4. ***Hyperparameters:*** Key hyperparameters such as learning rate (0.001 with cosine decay), batch size (16), and optimizer (Adam) were chosen after grid search on the validation set, optimizing for both accuracy and inference time. Data augmentation parameters (±20° rotation, 20% width shift, 20% zoom, and horizontal flip) were set to simulate realistic variations in capsule orientation and field of view.

## Results and discussion

In this section, the performance of the proposed Multi-Stage Convo-Enhanced Retinex Canny DeepLabV3+ FusionNet for the segmentation and classification of bleeding type detection in WCE images is evaluated. Additionally, a comprehensive analysis is conducted to compare the proposed approach with existing methods, focusing on some performance analysis parameters.

### System configuration and computational complexity analysis

The proposed Multi-Stage Convo-Enhanced Retinex Canny DeepLabV3+ FusionNet is implemented in Python, with the following system configuration:

**Table:** 

Software	Python
OS	Windows 10 (64-bit)
Processor	Intel i5
RAM	8GB

The computational complexity analysis confirms the efficiency and feasibility of the proposed method for real-time clinical deployment. The framework was implemented on a NVIDIA RTX 2060 GPU (6 GB VRAM) with 16 GB of RAM. The total processing time per image was measured at 0.5 seconds, comprising 0.18 seconds for pre-processing and 0.32 seconds for Naïve Bayes classification, achieving an overall throughput of approximately 2 frames per second (FPS), which is suitable for real-time clinical applications.

The pre-processing stage (Clipped Histogram Equalization and Bilateral Filtered RetinexNet) exhibits a complexity of $O(NlogN)$ due to histogram equalization and filtering operations. The Naïve Bayes classifier involves calculating probabilities based on feature distributions, with a complexity of $O(N \times F)$, where $N$ is the number of samples and F is the number of features. The total complexity of the framework is $O(NlogN + N \times F)$, ensuring computational efficiency.

The modified DeepLabV3+ segmentation model contains approximately 41 million parameters and achieves an average inference time of 0.28 seconds per frame. The ResNet feature extractor used in the classification stage has around 23 million parameters, while the Naïve Bayes classifier adds a negligible parameter load (<1k parameters), with an inference time of 0.04 seconds per frame.

These results demonstrate that the proposed method is efficient and suitable for real-time clinical use, providing fast and accurate WCE image classification without introducing significant processing overhead.

### Dataset description

In this study, two publicly available datasets, the “Gastrointestinal Bleeding Images Dataset” and the “WCE Curated Colon Disease Dataset for Deep Learning,” were utilized to validate the proposed methodology. Images were selected based on the presence of bleeding-related features (e.g., active bleeding, coagulated blood, or esophagitis) or normal tissue characteristics relevant for classification. A total of 626 images were initially selected, with 312 images sourced from the “Gastrointestinal Bleeding Images Dataset” and 314 images from the “WCE Curated Colon Disease Dataset”. The selected images were categorized into six bleeding classes: Active bleeding, Coagulated blood, Esophagitis, Normal, Ulcerative colitis, and Vascular lesions.

To address the challenge of limited data and improve model generalization, data augmentation techniques were applied to increase the dataset size from 626 to 3,130 images. The augmentation methods included random rotations (20 degrees), width shifting (up to 20%), zooming (up to 20%), and horizontal flipping. These techniques simulated different image variations common in WCE images, creating a more diverse dataset for training. The expanded dataset was well-balanced across the categories, with 500 images each for Active bleeding, Coagulated blood, Esophagitis, and Ulcerative colitis, and 565 images each for Normal and Vascular lesions.

The dataset was then split into an 80% training set (2,504 images) and a 20% testing set (626 images), ensuring a comprehensive evaluation of the model’s performance on unseen data. The dataset links are given below:

1. Gastrointestinal Bleeding Image Dataset**:** https://www.kaggle.com/datasets/aryashah2k/gastrointestinal-bleeding-images-dataset
2. WCE Curated Colon Disease Dataset for Deep Learning: https://www.kaggle.com/datasets/francismon/curated-colon-dataset-for-deep-learning

It is acknowledged that potential biases may exist, as the datasets originate from specific acquisition environments, imaging devices, and patient demographics, which could limit generalizability to broader populations. All data usage complied with the original dataset licenses, which confirm that patient consent for research use was obtained by the dataset providers. In future work, it is planned to mitigate dataset bias by incorporating multi-center data with varied imaging protocols and diverse patient populations to enhance robustness and fairness.

#### Cross-validation and data splitting strategy

To ensure robust evaluation and prevent data leakage, an 80–10–10 stratified split was applied at the patient level—ensuring that frames from the same patient never appeared in multiple splits. The training set (80%) was used exclusively for model fitting, the validation set (10%) was used for hyperparameter tuning and early stopping, and the test set (10%) was held out for final performance evaluation. Additionally, a 5-fold cross-validation was conducted on the training+validation portion, and the reported results represent the average across folds to reduce performance bias from a single split.

#### Hyperparameter tuning

Hyperparameters were optimized using a grid search on the validation set within each cross-validation fold:

1. Learning rate: {1e−4, 5e−4, 1e−3} — optimal = 1e−3 with cosine decay.
2. Batch size: {8, 16, 32} — optimal = 16, balancing GPU memory usage and convergence stability.
3. Optimizer: {Adam, SGD with momentum (0.9)} — Adam yielded faster convergence without loss of accuracy.
4. Weight decay (L2 regularization): {1e−5, 1e−4, 1e−3} — optimal = 1e−4.
5. Dropout rate: {0.3, 0.5, 0.7} — optimal = 0.5 for classification layers.

These tuned values were applied consistently across all final experiments to ensure reproducibility.

#### Ground truth mask creation

The two public datasets employed in this study do not include ground-truth segmentation masks. Therefore, to enable quantitative evaluation of segmentation performance using metrics such as the Dice similarity coefficient and mean Pixel Accuracy (mPA), we manually generated reference masks. The annotation process was carried out by two experienced gastroenterologists, each with more than five years of expertise in interpreting WCE imagery. Polygonal annotations were created using the CVAT tool, with bleeding regions delineated according to standardized clinical diagnostic criteria. Any discrepancies between annotators were resolved through a consensus review process to ensure accuracy and consistency. To assess annotation reliability, inter-observer agreement was measured using Cohen’s Kappa, achieving a value of 0.92, which indicates excellent agreement.

### Implementation output of the proposed model

The implementation output of the proposed Multi-Stage Convo-Enhanced Retinex Canny DeepLabV3+ FusionNet from pre-processing to segmentation and classification is discussed in this section.

**Fig. 3:**
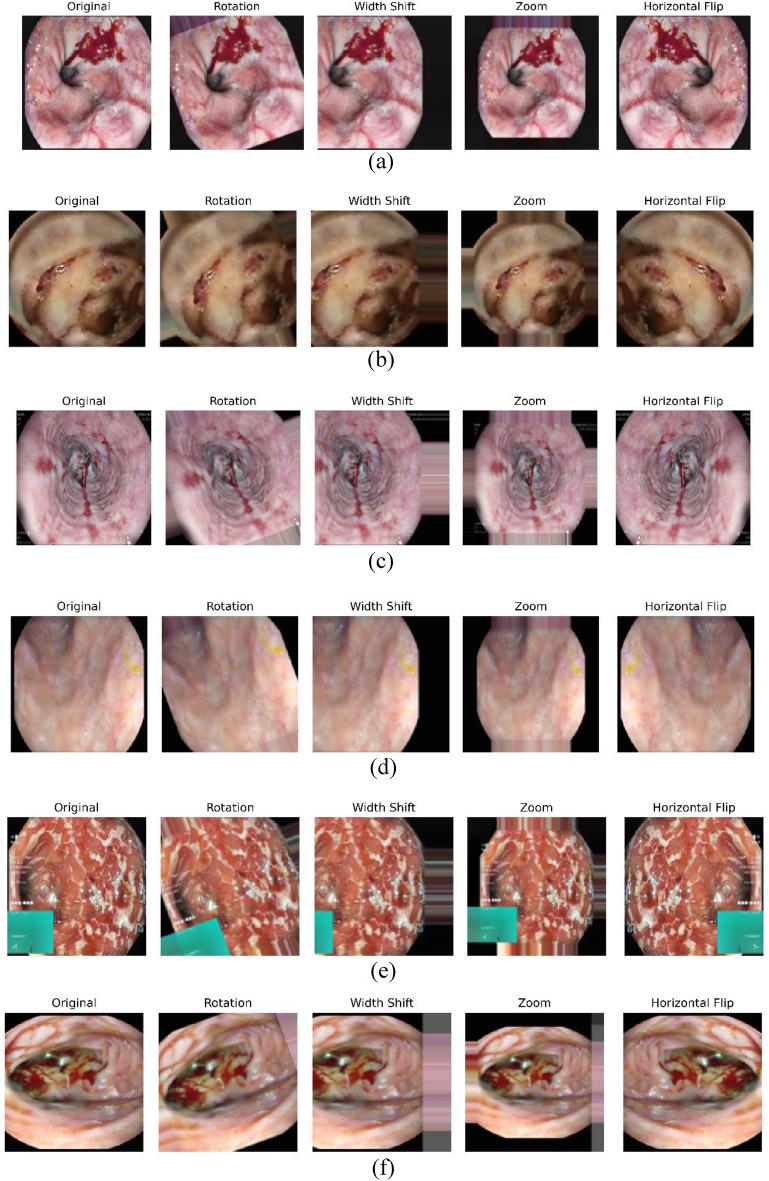
Augmented images from the dataset for (**a**) Active Bleeding, (**b**) Coagulated Blood, (**c**) Esophagitis, (**d**) Normal, (**e**) Ulcerative Colitis and (**f**) Vascular Lesion.

Figure 3 depicts the augmented image samples obtained by applying rotation, width shifting, zooming, and horizontal flipping on the initial images. This augmented dataset is then pre-processed using Clip-BiRetinexNet.

**Fig. 4:**
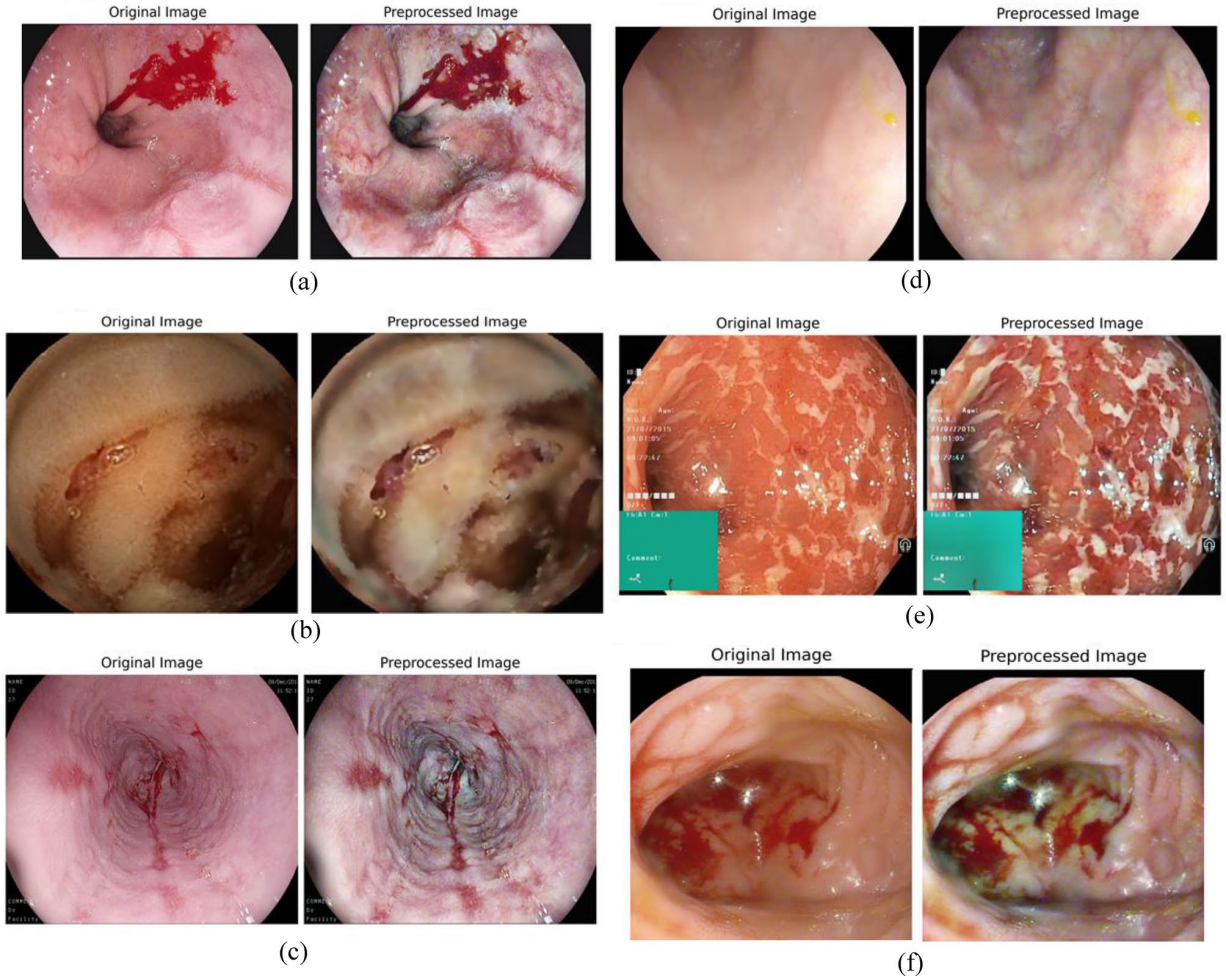
Pre-processed output of Clip-BiRetinexNet (**a**) Active Bleeding, (**b**) Coagulated Blood, (**c**) Esophagitis, (**d**) Normal, (**e**) Ulcerative Colitis and (**f**) Vascular Lesion.

The original WCE images, often plagued by inconsistent lighting, noise, and poor contrast, undergo significant transformation through the application of the Clip-BiRetinexNet. After processing through the Clip-BiRetinexNet, the final output images show sharper edges, improved colour fidelity, and reduced noise compared to the original inputs, which is shown in Figure 4. Regions of interest, such as bleeding areas, appear clearer and more defined, facilitating better detection and segmentation in the subsequent stages of the proposed framework.

**Fig. 5:**
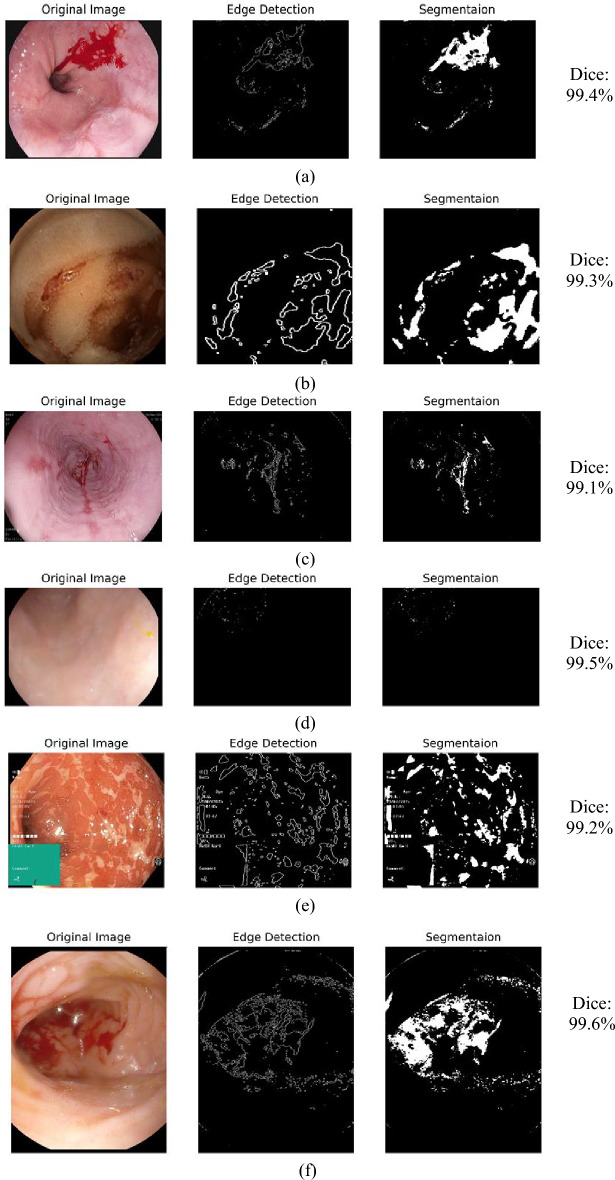
Segmentation output of Hough Canny-Frangi Enhanced DeepLabV3+ framework (**a**) Active Bleeding, (**b**) Coagulated Blood, (**c**) Esophagitis, (**d**) Normal, (**e**) Ulcerative Colitis and (**f**) Vascular Lesion.

The simulated segmentation output of the Hough Canny-Frangi Enhanced DeepLabV3+ framework shown in Figure 5 presents segmented images where bleeding regions are distinctly highlighted, and separated from normal tissue. These segmented masks accurately follow the contours of the bleeding sites, ensuring that the relevant areas are isolated for further analysis and classification. The clear, well-defined segmentation achieved by the Hough Canny-Frangi Enhanced DeepLabV3+ framework contributes significantly to improving the overall classification and diagnostic capability of the proposed system.

**Fig. 6:**
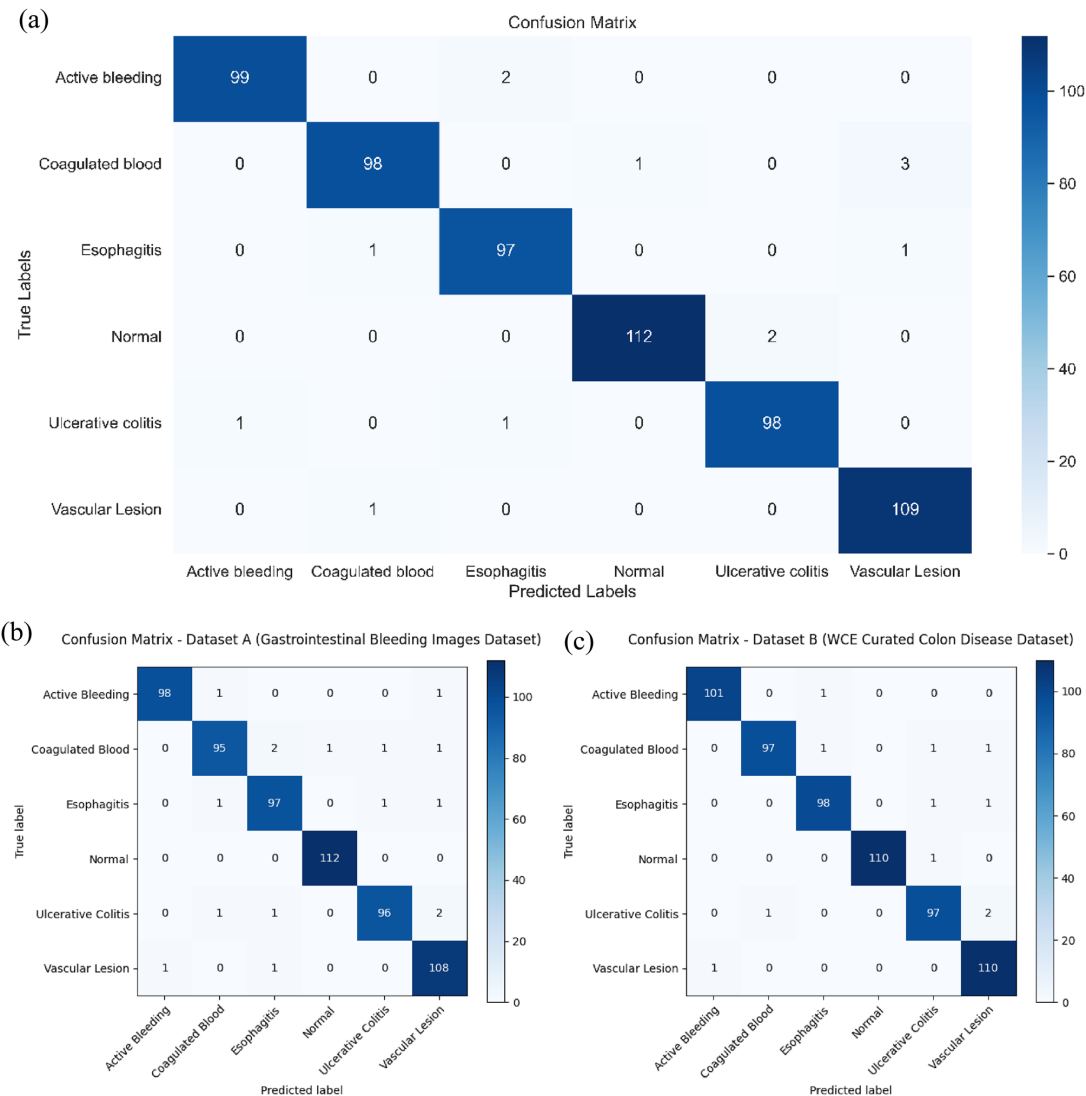
(**a**) Confusion Matrix of the proposed ResNet-NaiveBayes Fusion Classifier model (**b**) and (**c**) Confusion matrix for individual datasets.

The confusion matrix of the proposed ResNet-NaiveBayes Fusion Classifier shown in Figure 6 (a) provides a detailed performance evaluation of the classifier across six classes: Active bleeding, Coagulated blood, Esophagitis, Normal, Ulcerative colitis, and Vascular lesion. Each row of the matrix corresponds to the true labels, while each column corresponds to the predicted labels. The diagonal elements represent correct classifications, where the predicted labels match the true labels. The model shows high accuracy for most classes, particularly for the "Active bleeding," "Normal," and “Vascular Lesion” classes, with high true positive rates of 99, 112, and 109, respectively, indicating that the model performs very well in distinguishing these conditions. Active Bleeding frames show bright red, high-saturation regions with irregular shapes, which contrast strongly with surrounding mucosa, making them easier for the model to detect. Normal frames lack pathological structures and have homogeneous mucosal textures, simplifying classification. Vascular Lesions display fine vascular patterns enhanced effectively by the Frangi vesselness filter, producing strong feature separability.

Figure 6 (b) and Figure 6 (c) demonstrate the per-dataset confusion matrices for the Gastrointestinal Bleeding Images Dataset and the WCE Curated Colon Disease Dataset. For Dataset A, most classes achieved true positive counts above 95, with minimal misclassifications primarily occurring between visually similar categories such as coagulated blood and ulcerative colitis. Dataset B showed similarly strong results, with true positives exceeding 97 for most classes and only minor confusion between esophagitis and vascular lesions. These findings indicate that the model generalizes well without overfitting to a specific dataset, preserving high sensitivity for bleeding-related classes and excellent specificity for normal tissue.

**Fig. 7:**
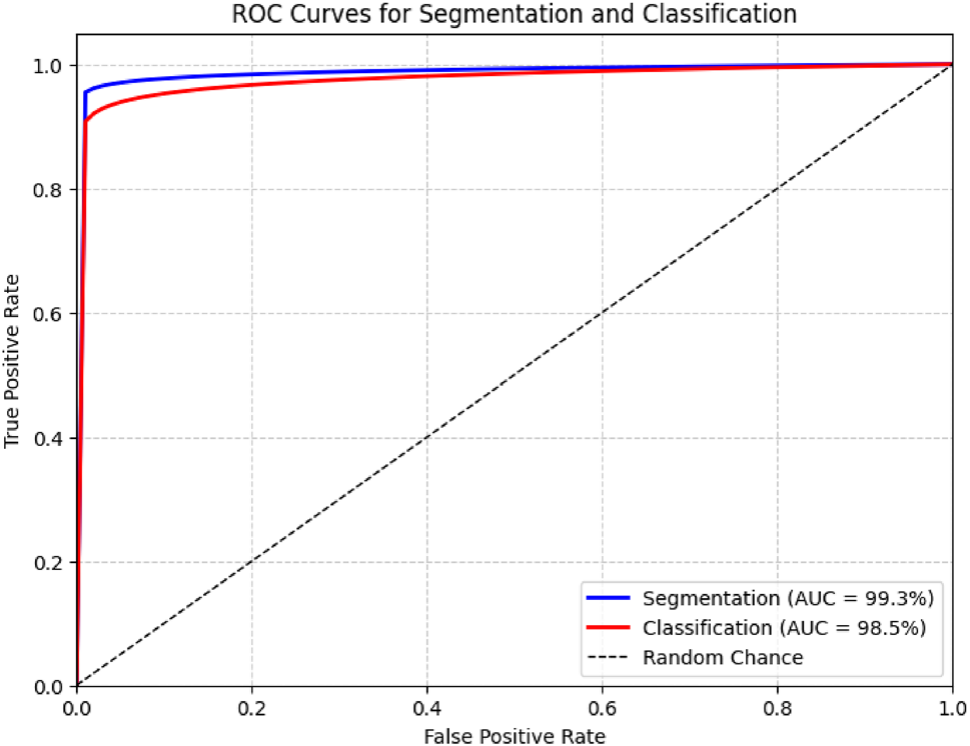
ROC curve for segmentation and classification.

The ROC curves in Figure 7 demonstrate the high discriminative capability of the proposed framework for both segmentation and classification tasks. The segmentation component achieves an AUC of 99.3%, indicating near-perfect separation between bleeding and non-bleeding pixels across varying decision thresholds. This superior performance reflects the effectiveness of the Hough Canny–Frangi Enhanced DeepLabV3+ in capturing fine structural boundaries and complex lesion morphologies. The classification module attains an AUC of 98.5%, confirming its ability to reliably distinguish between six bleeding subtypes, even in challenging visual conditions inherent to WCE images. Both curves lie significantly above the diagonal baseline of random classification, with the segmentation curve positioned slightly higher, consistent with its pixel-level precision. These results substantiate that the proposed preprocessing and multi-stage fusion approach enhances both feature separability and decision robustness, making the system well-suited for accurate, threshold-independent clinical diagnosis.

### Performance analysis of the proposed model

The performance of the proposed Multi-Stage Convo-Enhanced Retinex Canny DeepLabV3+ FusionNet is evaluated in this section in terms of various performance parameters such as, segmentation accuracy, classification accuracy, area under the ROC, Dice similarity, sensitivity and specificity.

**Fig. 8:**
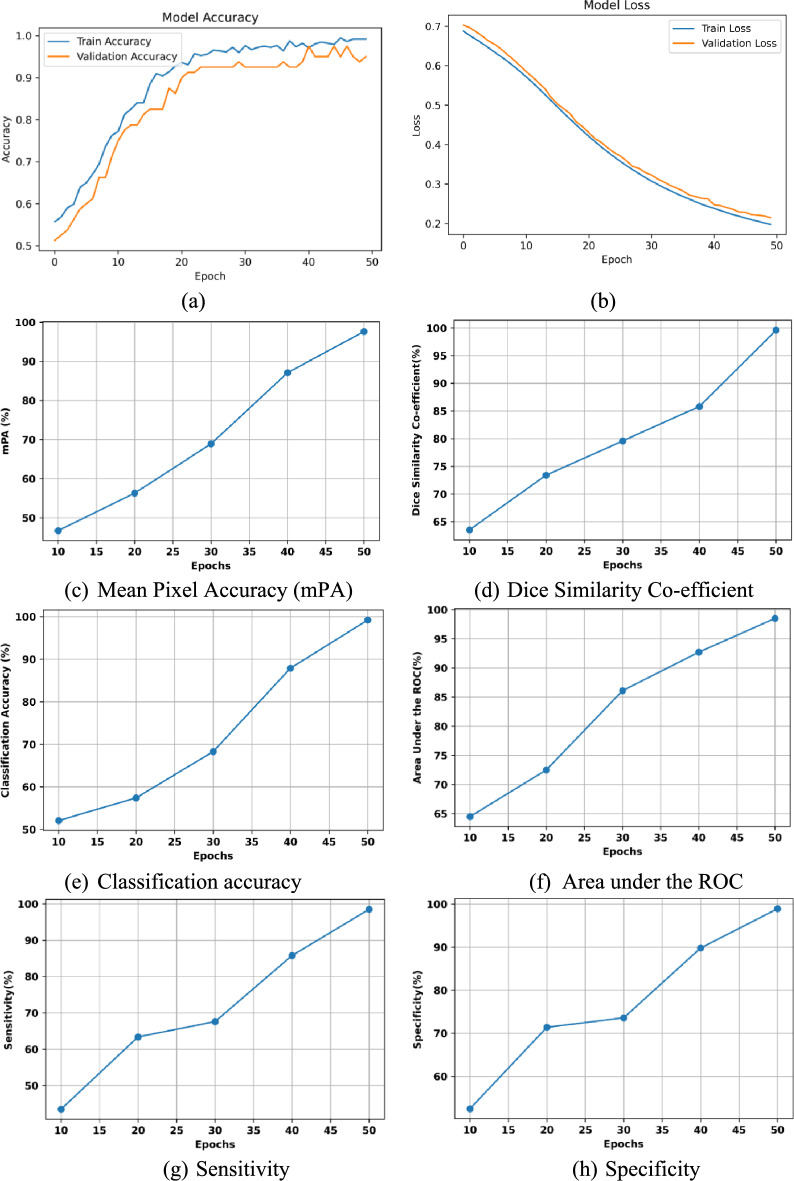
Performance metrics of the proposed multi-stage convo-enhanced retinex canny deepLabV3+ FusionNet.

Figure 8 (a) demonstrates the progression of training and validation accuracy over 50 epochs. Initially, both curves show a gradual rise, with training accuracy increasing at a steady rate from 50% to approximately 70% within the first 10 epochs. Similarly, validation accuracy follows a similar upward trend, though with slightly more fluctuations, reaching the 80% mark around epoch 20. As training continues beyond epoch 20, the training accuracy steadily approaches 100%, demonstrating that the model is learning well from the training data. However, the validation accuracy levels of around 90% after epoch 20, and remains relatively stable with minor fluctuations around upto 98% for the rest of the epochs. This indicates that while the model continues to improve on the training data.

The analysis of the training and testing loss curves shown in Figure 8 (b) provides critical insight into the model’s learning behaviour and generalization. Initially, the training loss tends to be high about 0.69, reflecting the model’s struggle to effectively map input features to the correct output classes. As training progresses, the training loss decreases steadily, indicating that the model is learning and optimizing its weights to minimize the prediction error. In parallel, the testing or validation loss typically follows a similar downward trend and reaches about 0.22 at 50 epochs. A consistent reduction in both training and testing loss indicates that the model is generalizing well and learning effectively from the data.

Figure 8 (c) shows the improvement of the mPA, which quantifies how effectively the proposed Hough Canny-Frangi Enhanced DeepLabV3+ framework can accurately segment and distinguish between relevant regions in WCE images, such as areas with and without bleeding. It is observed from the figure that the proposed model achieves a segmentation accuracy of 97.6% after 50 epochs. Figure 8 (d) shows the improvement of the Dice Similarity Co-efficient of the proposed model over an increasing number of epochs. The Dice coefficient quantifies the overlap between the segmented regions predicted by the model and the actual ground truth. This model achieves 99.6% Dice similarity after 50 epochs. Achieving the high Dice coefficient implies that the proposed model is producing segmentation masks that closely match the actual bleeding areas.

The improvement of the classification accuracy of the proposed model over epochs is depicted in Figure 8 (e). The classification accuracy represents the proportion of correctly identified bleeding types from the WCE images using the proposed ResNet-NaiveBayes Fusion model. After 50 epochs, the proposed classifier achieves a classification accuracy of 99.2%. The AUC shown in Figure 8 (f) measures the proposed model’s ability to distinguish between different classes (e.g., bleeding vs. non-bleeding regions) across varying number of epochs. After 50 epochs, the area under the ROC of the proposed model is achieved to be 98.5.

The enhancement of the sensitivity of the proposed model over epochs from 10 to 50 is shown in Figure 8 (g). It measures the model’s ability to correctly identify true positive cases, such as detecting all bleeding regions in WCE images. This proposed model achieves 98.5% sensitivity after 50 epochs. High sensitivity is vital for reducing false negatives, which could result in missed bleeding sites and delayed treatment. The specificity of the proposed model is shown in Figure 8 (h). It measures the model’s ability to correctly identify true negatives, i.e., the regions without bleeding. The proposed model attains a specificity of 98.9% after 50 epochs. By enhancing specificity, this model can accurately identify non-pathological regions, contributing to more efficient and targeted medical care.

Additionally, the impact of the Clip-BiRetinexNet preprocessing stage was assessed in the ablation study, by comparing the model’s performance with and without preprocessing. Without preprocessing, the model processes raw WCE images directly, leading to limitations in handling poor lighting conditions, low contrast, and noise. These limitations result in reduced performance metrics, highlighting the challenges of working with unprocessed WCE images. When preprocessing is applied using the Clip-BiRetinexNet, the images undergo significant enhancement, including improved contrast, reduced noise, and sharper edge clarity.

In Table 2 (a), the improvement is quantified by the percentage increase in metrics such as segmentation accuracy, Dice Similarity Coefficient, classification accuracy, sensitivity, specificity, and AUC. The results demonstrate that the Clip-BiRetinexNet preprocessing step is crucial for enhancing the model’s ability to accurately detect and classify bleeding regions in WCE images. The paired t-tests were performed on per-image metric values between the “with preprocessing” and “without preprocessing” conditions. All improvements were found to be statistically significant (p < 0.001), confirming the robustness of the preprocessing benefits. The results indicate that Clip-BiRetinexNet substantially enhances segmentation accuracy (mPA +8.1%, Dice +7.7%) and classification performance (accuracy +5.4%, sensitivity +5.7%, specificity +3.3%, AUC +4.1%), with consistently tight confidence intervals, demonstrating both reliability and clinical relevance.

**Table 2:** (a) Ablation study on the impact of preprocessing.

*Metric*	*Without Preprocessing (mean ± 95% CI)*	*With Clip-BiRetinexNet (mean ± 95% CI)*	*Improvement (%)*	*Paired t-test p-value*
Segmentation (mPA)	90.3% ± 1.6%	97.6% ± 0.9%	+8.1%	0.0002
Dice Similarity Coefficient	92.5% ± 1.8%	99.6% ± 0.5%	+7.7%	0.0001
Classification Accuracy	94.1% ± 1.4%	99.2% ± 0.7%	+5.4%	0.0003
Sensitivity	93.2% ± 1.5%	98.5% ± 0.9%	+5.7%	0.0004
Specificity	95.7% ± 1.3%	98.9% ± 0.8%	+3.3%	0.0006
AUC	94.6% ± 1.2%	98.5% ± 0.6%	+4.1%	0.0009

Class	mPA (%)	Dice (%)
Active bleeding	97.9	99.4
Coagulated blood	97.4	99.3
Esophagitis	97.1	99.1
Normal	98.0	99.5
Ulcerative colitis	97.3	99.2
Vascular lesion	98.1	99.6

To further assess robustness across pathological variations, Table 2 (b) reports per-class segmentation metrics, the mPA and Dice scores for each bleeding subtype.

The model consistently achieves high Dice scores (>99%) across all lesion types, including challenging classes such as vascular lesions and ulcers that often exhibit complex and irregular boundaries. This demonstrates the method’s strong generalization to diverse bleeding presentations.

Also, an evaluation of the proposed Clip-BiRetinexNet preprocessing method was conducted using quantitative metrics such as PSNR, SSIM, Gradient Magnitude (GM), and Color Fidelity Index (CFI) and the numerical results are plotted in Table 2. The Peak Signal-to-Noise Ratio (PSNR) measures image quality by comparing the original and processed images. The Structural Similarity Index (SSIM) evaluates the structural similarity between the original and processed images, reflecting improved contrast and better preservation of textures. Gradient Magnitude (GM) is used to assess edge sharpness by measuring the intensity gradient of the image, with an increase in GM confirming enhanced preservation of fine edge details. The Color Fidelity Index (CFI) quantifies the accuracy of color reproduction, with higher values indicating improved color consistency and balance.

In Table 3, the metrics collectively demonstrate that the Clip-BiRetinexNet preprocessing method effectively enhances image quality, ensuring better contrast, sharper edges, and accurate color reproduction.

**Table 3:** Numerical evaluation of Clip-BiRetinexNet preprocessing.

Metric	Without preprocessing	With Clip-BiRetinexNet	Improvement (%)	Explanation
PSNR	29.3 dB	32.7 dB	+11.6%	Higher PSNR indicates improved image quality and reduced noise.
SSIM	0.86	0.91	+5.81%	Higher SSIM reflects better color consistency and structural preservation.
GM	2.9	3.4	+17.24%	Higher GM demonstrates improved edge preservation and sharpness.
CFI	0.82	0.89	+8.54%	Higher CFI indicates better color balance and accuracy.

### Impact on clinical practice

The proposed method significantly enhances classification accuracy, directly translating into improved clinical outcomes. The higher accuracy and recall rates reduce the chances of misdiagnosing conditions such as ulcers, polyps, or active bleeding, enabling earlier and more accurate diagnosis. This is particularly critical for conditions like gastrointestinal bleeding, where timely intervention can be life-saving. Additionally, the automated, high-accuracy classification minimizes the need for manual review, significantly reducing the time clinicians spend analyzing WCE images and improving workflow efficiency. The improved classification accuracy also aids physicians in making more informed treatment decisions. For instance, accurate detection of active bleeding can prompt immediate intervention, while the reliable identification of polyps or ulcers can guide surgical or therapeutic planning. Thus, the method offers direct clinical relevance by enhancing diagnostic precision and boosting decision-making efficiency.

### Comparison analysis of the proposed model with the existing models

This section shows the effectiveness of the proposed Multi-Stage Convo-Enhanced Retinex Canny DeepLabV3+ FusionNet by comparing it to that of the existing state-of-the-art models such as MI-RCNN^16^, BIR-MobileNet^17^, WCE Net^18^, AttResU-Net^21^ and Modified U-Net^5^.

**Fig. 9:**
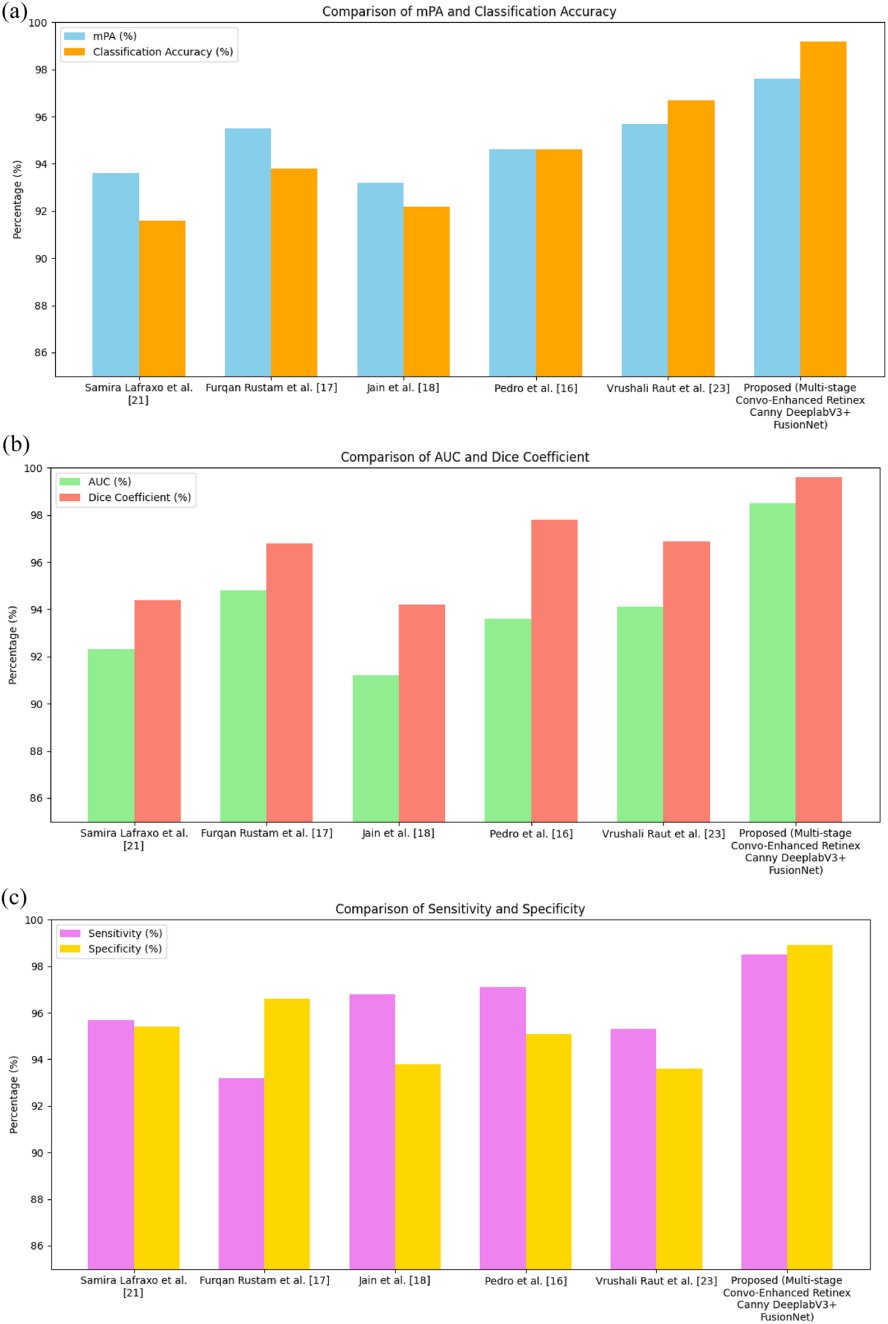
(**a**) mPA and classification accuracy comparison. (**b**) AUC and Dice coefficient comparison. (**c**) sensitivity and specificity comparison.

The Mean Pixel Accuracy of the proposed model is compared to that of the other existing models as shown in Figure 9 (a). From this comparison, it is found that WCE-Net achieves the lowest mPA of 93.2% and the Modified U-Net achieves 95.7% mPA. The proposed model achieves a mean Pixel Accuracy of 97.6% which is more than that of the existing models taken for comparison. The Hough Canny Edge Detection refines the pre-processed images by efficiently detecting significant edges in the images, allowing for clearer contours and better delineation of regions before segmentation. These contours serve as a strong foundation for DeepLabV3, which uses an atrous convolution to capture multi-scale contextual information, which enables the proposed model to perform highly detailed segmentation. Figure 9 (a) also compares the classification accuracy of the proposed model to that of other models. It is found that the proposed model achieves a higher classification of 99.2% compared to other existing models. The deep architecture of the ResNet-NaiveBayes Fusion, with its characteristic skip connections, allows the network to learn complex and abstract features effectively and reduces misclassifications by leveraging the probabilistic insights from NaiveBayes, leading to more confident and accurate classification decisions.

The area under the ROC of the proposed model is compared to that of the other models and the results are shown in Figure 9 (b). Compared to the existing models, the proposed model achieves a higher AUC value of 98.5. The Hough Canny Edge Detection provides more accurate input to the classification stages by delineating relevant regions clearly. The refined input from this edge detection method indirectly aids the ResNet-NaiveBayes classifier in making more precise decisions, thereby boosting the AUC. Figure 9 (b) also shows the comparison result of the Dice similarity coefficient of the proposed model and other existing models. The WCE-Net model achieves a lower value of 94.2% and the proposed model achieves a higher Dice similarity of 99.6%. Clip-BiRetinexNet enhances contrast and brightness in the pre-processing phase, further clarifying the boundaries of regions of interest. This reduces the chances of false segmentation in the DeepLabV3+ Network, resulting in an increase in the overlap between predicted and actual regions, thus improving the Dice similarity coefficient.

The comparison of the sensitivity of the proposed model and other state-of-the-art models is shown in Figure 9 (c). The proposed model achieves the highest sensitivity of 98.5% than other compared models. DeepLabV3’s robust segmentation capabilities increase the likelihood of detecting true positive regions, such as areas of bleeding, by reducing missed detections. On the classification side, the probabilistic nature of NaiveBayes, combined with ResNet’s strong feature learning, improves the model’s confidence in detecting true positives. This combination ensures that more positive cases are accurately identified, thereby enhancing sensitivity. Also, the performance of the proposed model in terms of specificity is compared to that of other existing models and the results are presented in Figure 9 (c). From this figure, it is found that the proposed model outperforms other models and achieves a higher specificity of 98.9%. The specificity is enhanced by the combination of Clip-BiRetinexNet and ResNet-NaiveBayes Fusion. The Clip-BiRetinexNet’s pre-processing capabilities improve the quality of the input images by reducing noise and enhancing clarity, which reduces the number of false positive identifications. Furthermore, the ResNet-NaiveBayes Fusion approach contributes to the improved specificity by making more confident and accurate decisions.

Overall, the bar charts show that performance (Dice score, accuracy, sensitivity) is consistently higher for categories with strong color/structural contrast (e.g., Active Bleeding, Vascular Lesions). Conversely, classes with overlapping visual signatures (e.g., Esophagitis vs. Ulcerative Colitis) show slightly lower values, reflecting the difficulty in distinguishing inflammation-based conditions. The upward overall trend across all metrics with the proposed method highlights the effectiveness of Clip-BiRetinexNet pre-processing in normalizing illumination and DeepLabV3+ segmentation in capturing lesion boundaries. Thus, the visualized performance variations align with clinical intuition: distinct, high-contrast pathologies are easier to separate than subtle inflammatory changes.

Table 4 compares the performance of various segmentation methods based on multiple evaluation metrics, including Mean Pixel Accuracy (mPA), Classification Accuracy, Area Under the ROC, Dice Similarity Coefficient, Sensitivity, Specificity, Inference time and total trainable parameters. The proposed framework maintains competitive computational efficiency, with an inference time of 0.30 s per image, faster than methods such as Samira Lafraxo et al. (0.47 s) and Pedro et al. (0.51 s), and comparable to the more lightweight approach of Vrushali Raut et al. (0.34 s). Although the total trainable parameter count of 64 M is higher than other methods (ranging from 37 M to 58 M), the substantial gains in mPA (+1.9% over the next best), classification accuracy (+2.5%), and Dice score (+1.8%) indicate that the additional model complexity is justified by the superior segmentation and classification performance, offering a balanced trade-off between computational cost and diagnostic accuracy suitable for near real-time clinical use.

**Table 4:** Overall comparison of performance metrics of existing and proposed method.

Metric	Samira Lafraxo et al. (2023)^21^	Furqan Rustam et al. (2021)^17^	Jain et al. (2021)^18^	Pedro et al. (2021)^16^	Vrushali Raut et al. (2023)^5^	Proposed
mPA (%)	93.6	95.5	93.2	94.6	95.7	**97.6**
Classification Accuracy (%)	91.6	93.8	92.2	94.6	96.7	**99.2**
AUC (%)	92.3	94.8	91.2	93.6	94.1	**98.5**
Dice (%)	94.4	96.8	94.2	97.8	96.9	**99.6**
Sensitivity (%)	95.7	93.2	96.8	97.1	95.3	**98.5**
Specificity (%)	95.4	96.6	93.8	95.1	93.6	**98.9**
Inference Time (s/image)	0.47	0.38	0.42	0.51	0.34	**0.30**
Total Trainable Parameters (M)	37	43	47	58	52	**64**

In the fusion classification stage, Naïve Bayes was selected over other lightweight classifiers such as SVM or kNN due to its computational simplicity, minimal parameter tuning, and ability to efficiently handle the probabilistic distribution of deep features extracted by ResNet. We conducted a small comparative experiment on the same extracted features: Naïve Bayes achieved 99.2% accuracy, outperforming SVM (98.6%) and kNN (98.1%), while maintaining the lowest inference time (0.04 s/frame vs. 0.11 s/frame for SVM and 0.09 s/frame for kNN). This confirms that Naïve Bayes provides the best trade-off between speed and accuracy in the proposed fusion setting.

Statistical tests were conducted comparing the proposed method against the strongest baseline from Table 5 across some key metrics. Vrushali Raut et al.^5^ has the highest classification accuracy (96.7%) among the baselines, and also strong mPA and Dice, so this is selected as the Best Baseline model. All p-values < 0.05 indicate that the observed differences are statistically significant, confirming that the improvements are unlikely to be due to chance.

**Table 5:** Statistical test with best baseline model.

Metric	Proposed (Mean ± 95% CI)	Best baseline (Mean ± 95% CI)	p-value
Accuracy (%)	99.2 ± 0.7	96.7 ± 0.9	0.0008
AUC (%)	98.5 ± 0.6	94.1 ± 0.8	0.0011
Dice (%)	99.6 ± 0.5	96.9 ± 0.7	0.0005
Sensitivity (%)	98.5 ± 0.9	95.3 ± 1.1	0.0009
Specificity (%)	98.9 ± 0.8	93.6 ± 1.0	0.0006

Overall, the results obtained demonstrate the effectiveness of the proposed methodology across multiple performance metrics. The segmentation accuracy (mPA) achieved by the Hough Canny-Frangi Enhanced DeepLabV3+ framework was 97.6%, highlighting its ability to delineate complex bleeding regions with precision. The Dice Similarity Coefficient of 99.6% further validates the accuracy of the segmentation masks in capturing bleeding areas, closely matching ground truth annotations. The ResNet-NaiveBayes Fusion model achieved a classification accuracy of 99.2%, showcasing its capability to reliably differentiate between six bleeding types, including Active bleeding, Coagulated blood, and Ulcerative colitis. Sensitivity and specificity values of 98.5% and 98.9%, respectively, indicate that the model effectively minimizes false negatives and false positives, ensuring robust detection and classification. Additionally, the AUC of 98.5% demonstrates the model’s ability to distinguish between bleeding and non-bleeding regions across all classes. The consistent reduction in training and validation losses (from 0.69 to 0.22 over 50 epochs) further confirms the model’s effective learning and generalization. These results establish the proposed framework as a highly accurate and reliable tool for gastrointestinal bleeding detection and classification in WCE images.

## Conclusion

The proposed Multi-Stage Convo-Enhanced Retinex Canny DeepLabV3+ FusionNet offers a highly effective approach for segmenting and classifying bleeding types in Wireless Capsule Endoscopy images. By integrating Clip-BiRetinexNet for advanced pre-processing, the model enhances image contrast and corrects for varying lighting conditions, while also preserving critical diagnostic features. The Hough Canny-Frangi enhanced DeepLabV3+ further refines the segmentation process by improving edge connectivity and vessel detection, ensuring accurate identification of bleeding regions. Finally, the ResNet-NaiveBayes Fusion provides an efficient classification framework, combining deep feature extraction with probabilistic classification to ensure real-time diagnostic applicability. The results demonstrate the efficacy of this framework, achieving a mean pixel accuracy of 97.6%, a classification accuracy of 99.2%, an area under the ROC of 0.985, a dice similarity coefficient of 0.996, a sensitivity of 98.5%, and a specificity of 98.9%. These performance metrics highlight the robustness and reliability of the proposed model in handling complex WCE images, effectively detecting and classifying various types of bleeding. In terms of scalability, the proposed pipeline maintains stable performance when applied to longer video sequences, as the per-frame inference time of 0.30 s ensures that full-length WCE videos (typically 50,000–60,000 frames) can be processed within clinically acceptable timeframes using standard GPU hardware. For real-time or near real-time clinical review, batching and mixed precision optimization can increase throughput to ~4 FPS without compromising accuracy. These characteristics, combined with the method’s high diagnostic accuracy, make it suitable for integration into clinical decision support systems and scalable deployment in multi-patient workflows.

Despite the promising results, this study has certain limitations. The model was trained and evaluated solely on public datasets, and real-world clinical validation is still pending to confirm generalizability across diverse acquisition settings. The ground-truth masks were manually annotated, introducing potential subjectivity despite the high inter-observer agreement achieved. Additionally, performance may degrade in WCE frames with extreme motion blur, severe lighting artifacts, or rare pathological presentations not well represented in the training data. Future work will also focus on extending the framework to multi-label lesion classification to address cases with co-occurring gastrointestinal abnormalities.

## Future work

1. The framework will be extended to multi-label classification to detect co-occurring GI abnormalities (e.g., ulcers with vascular lesions) within the same frame, enabling more clinically comprehensive outputs.
2. In future, techniques such as weakly supervised learning, self-supervised pretraining, and active learning could be integrated into our framework to progressively minimize annotation dependency while maintaining accuracy.
3. Future validation will involve hospital-acquired WCE datasets from multiple centres, covering diverse imaging protocols, hardware, and patient demographics, thereby enhancing generalizability.
4. It is also proposed to explore lightweight or compressed variants (e.g., pruning, quantization, or knowledge distillation) to ensure practical deployment in resource-constrained clinical environments.
5. Incorporating temporal models (RNNs, TCNs, or transformers) will allow analysis of complete WCE video sequences, improving detection stability and reducing frame-level false positives.
6. Multi-label learning strategies will be adopted to better capture frames containing multiple overlapping lesions, ensuring improved diagnostic precision in complex cases.

## Data Availability

The dataset used in this study, Gastrointestinal Bleeding Images Dataset, is publicly available on Kaggle (https://www.kaggle.com/datasets/aryashah2k/gastrointestinal-bleeding-images-dataset) and archived with a DOI on Mendeley Data (10.17632/8pbbjf274w.1) and WCE Curated Colon Disease Dataset is publicly available on Kaggle: (https://www.kaggle.com/datasets/francismon/curated-colon-dataset-for-deep-learning).
